# Histone Deacetylases as Modulators of the Crosstalk Between Skeletal Muscle and Other Organs

**DOI:** 10.3389/fphys.2022.706003

**Published:** 2022-02-18

**Authors:** Alessandra Renzini, Marco D’Onghia, Dario Coletti, Viviana Moresi

**Affiliations:** ^1^Unit of Histology and Medical Embryology, Department of Anatomy, Histology, Forensic Medicine and Orthopedics, Sapienza University of Rome, Rome, Italy; ^2^Biological Adaptation and Ageing, Institut de Biologie Paris-Seine, Sorbonne Université, Paris, France; ^3^Institute of Nanotechnology (Nanotec), National Research Council, Rome, Italy

**Keywords:** HDACs, tissue crosstalk, soluble factors, epigenetics, HDAC inhibitors (HDACi)

## Abstract

Skeletal muscle plays a major role in controlling body mass and metabolism: it is the most abundant tissue of the body and a major source of humoral factors; in addition, it is primarily responsible for glucose uptake and storage, as well as for protein metabolism. Muscle acts as a metabolic hub, in a crosstalk with other organs and tissues, such as the liver, the brain, and fat tissue. Cytokines, adipokines, and myokines are pivotal mediators of such crosstalk. Many of these circulating factors modulate histone deacetylase (HDAC) expression and/or activity. HDACs form a numerous family of enzymes, divided into four classes based on their homology to their orthologs in yeast. Eleven family members are considered classic HDACs, with a highly conserved deacetylase domain, and fall into Classes I, II, and IV, while class III members are named Sirtuins and are structurally and mechanistically distinct from the members of the other classes. HDACs are key regulators of skeletal muscle metabolism, both in physiological conditions and following metabolic stress, participating in the highly dynamic adaptative responses of the muscle to external stimuli. In turn, HDAC expression and activity are closely regulated by the metabolic demands of the skeletal muscle. For instance, NAD+ levels link Class III (Sirtuin) enzymatic activity to the energy status of the cell, and starvation or exercise affect Class II HDAC stability and intracellular localization. SUMOylation or phosphorylation of Class II HDACs are modulated by circulating factors, thus establishing a bidirectional link between HDAC activity and endocrine, paracrine, and autocrine factors. Indeed, besides being targets of adipo-myokines, HDACs affect the synthesis of myokines by skeletal muscle, altering the composition of the humoral milieu and ultimately contributing to the muscle functioning as an endocrine organ. In this review, we discuss recent findings on the interplay between HDACs and circulating factors, in relation to skeletal muscle metabolism and its adaptative response to energy demand. We believe that enhancing knowledge on the specific functions of HDACs may have clinical implications leading to the use of improved HDAC inhibitors for the treatment of metabolic syndromes or aging.

## Introduction

Skeletal muscle significantly affects body mass and energy consumption, being characterized by a highly dynamic response to energy demand (Baskin et al., 2015). Indeed, skeletal muscle is the most significant metabolic organ, accounting for up to 30% of resting energy expenditure (Zurlo et al., 1990). Considered a key site for glucose uptake and storage, and a reservoir of amino acids, skeletal muscle exerts a crucial role in modulating energy and protein metabolism throughout the body (Turner et al., 2014; Ahima and Park, 2015). Adult skeletal muscles have a diverse pattern of myofibers (Staron, 1997): the slow-twitch, or type I, myofibers are characterized by a higher content of mitochondria and have an oxidative metabolism, as opposed to the fast-twitch, or type II, myofibers, which mostly rely on a glycolytic metabolism (Schiaffino and Reggiani, 2011; Talbot and Maves, 2016). Metabolic demand or a disease state both affect the composition of the skeletal muscle fiber type. For instance, insulin resistance correlates with an increase in the anaerobic and glycolytic capacities of muscle, similarly to what happens in obesity and diabetes (Simoneau et al., 1995). On the other hand, age-related sarcopenia is characterized by an increase in the relative amount of slow-twitch fibers and by the atrophy of the fast ones (Gannon et al., 2009; Daou et al., 2020; Berardi et al., 2021).

The mechanisms whereby skeletal muscle mediates metabolic changes in other tissues is a clinically relevant, dynamic avenue of investigation. An increasingly high number of muscle-derived soluble molecules is known to induce a plethora of effects in the whole body and to mediate inter-organ cross-talk (Severinsen and Pedersen, 2020) and numerous studies also reveal that changes in skeletal muscle factors can modulate metabolism systemically (Chen et al., 2010; Correia et al., 2015; Pereira et al., 2017). The expression of many muscle-derived soluble factors is reported as being altered in disease, including in cancer, type 2 diabetes, obesity, or heart failure (Liu et al., 2015; Berezin et al., 2021; Evans et al., 2021; Pin et al., 2021), suggesting the importance of these factors in the crosstalk between muscle and other organs/tissues in pathological conditions. However, the metabolic adaptations of the skeletal muscle, up to now often merely seen as a consequence of a disease state, may be exploited in a pro-active way to treat metabolic disorders. In this scenario, exercise training as a preventive and therapeutic strategy in several diseases has already been proposed (Leal et al., 2018; de Lima et al., 2020; Severinsen and Pedersen, 2020). Indeed, muscle contraction represents a major physiological stimulus to activate the release of a variety of soluble factors, in a time- and intensity-dependent fashion (Hutchinson et al., 2019; Kurgan et al., 2019; Piccirillo, 2019; Florin et al., 2020).

Histone deacetylases (HDACs) work as epigenetic factors by repressing gene transcription through the removal of acetyl groups from the tails of histone proteins or, alternatively, modulate various cellular responses through their deacetylase activity on non-histone proteins (Glozak et al., 2005; Hyndman and Knepper, 2017). We find it intriguing that HDACs modify gene expression in the skeletal muscle tissue in response to humoral factors, and that, in turn, several HDACs modulate the expression of soluble factors, ultimately affecting their levels in the circulation. The fact that the genetic expression of secreted products is regulated by epigenetic factors adds up to the complexity of the regulatory network controlling myokine production. Also, that multiple tissues are responsible for the coordinated production of the same endocrine factors, such as certain adipo-myokines, highlights this phenomenon in all its complexity.

In this review article, first we introduce HDACs (paragraph 2) and their key role in skeletal muscle metabolic responses (paragraph 3), then we review the most recent findings on how myokines directly or indirectly regulate metabolic reprogramming (paragraph 4): in particular, we focus on those myokines that affect HDAC expression and activity (paragraph 5) and, vice-versa, on those HDACs that affect myokine synthesis and release (paragraph 6); lastly, we conclude with the clinical implications of the use of HDAC inhibitors (paragraph 7).

## The Histone Deacetylases and Their Classification

Chromatin is a highly organized nucleoprotein complex, mainly composed by DNA and histone proteins that form nucleosomes. The DNA transcription rate depends, among other factors, on changes in the structure of the nucleosomes. Highly compact and dense chromatin, known as heterochromatin, is associated with the repression of transcriptional activity; whereas open chromatin, or euchromatin, is associated with transcriptional activation. The packaging of chromatin depends mainly on modifications, including acetylation, of the N-terminal histone tails, which protrude from the nucleosome. Histone acetylation levels are regulated through the interplay between two families of enzymes, namely histone acetyltransferases (HATs) and HDACs, and chromatin (Peserico and Simone, 2011). The deacetylation of histones increases their positive charge and consequently their affinity to the DNA, inducing the formation of a compacted, transcriptionally repressed chromatin structure. However, HDACs also target non-histone proteins and finely tune their activity and function by means of deacetylation (Bannister and Kouzarides, 2011; Peserico and Simone, 2011; Hyndman and Knepper, 2017).

To date, 18 different mammalian HDACs have been identified and divided into four classes according to their sequence similarity to yeast counterparts (Seto and Yoshida, 2014; Park and Kim, 2020): Class I HDACs (HDAC1, 2, 3, and 8), Class II HDACs (HDAC4, 5, 6, 7, 9, and 10), Class III HDACs (Sirtuins), which differ from the others because they use NAD^+^ as a cofactor rather than Zn^2+^, and Class IV HDACs (HDAC11). Class II HDACs is further divided into two subgroups, Class IIa (HDAC4, 5, 7, and 9) and Class IIb HDACs (HDAC6 and 10), which differ significantly in many biological aspects, from subcellular localization to their involvement in diseases. Whereas most Class I HDACs are ubiquitously expressed and localized in the nucleus, the Class II HDACs are characterized by tissue-specific expression and stimulus-dependent nucleus-to-cytoplasm shuttling (Haberland et al., 2009). Class III HDACs, which are ubiquitously expressed in human tissues (Frye, 1999), have differential subcellular localizations: SIRT1, SIRT6, and SIRT7 are primarily detected in the nucleus, SIRT2 is mainly found in the cytosol, while SIRT3, SIRT4, and SIRT5 are present exclusively in mitochondria (Michishita et al., 2005). HDAC11, the only member of Class IV, seems to be mainly expressed in the kidney, heart, brain, skeletal muscle, and testis, and is localized in the nucleus of the cell (Michishita et al., 2005).

Histone deacetylases control essential phenomena such as cell cycle progression, cell survival and differentiation (Moresi et al., 2016). Consistently, HDAC deregulation plays a key role in many pathologies, including cancer, neurological, inflammatory and cardiac diseases, as well as metabolic and neuromuscular disorders (Haberland et al., 2009; Volmar and Wahlestedt, 2015; Li and Seto, 2016; Pigna et al., 2019). The involvement of HDACs in the regulation of skeletal muscle metabolism is highlighted below.

## Histone Deacetylases Are Key Regulators of Skeletal Muscle Metabolism

The interplay between epigenetics and skeletal muscle metabolism has been thoroughly described (Sharples et al., 2016; Tzika et al., 2018; Jacques et al., 2019). In particular, HDAC activity orchestrates the metabolic reprogramming of skeletal muscle in response to physiological challenges and in several disease states (Howlett and McGee, 2016; Bianchi et al., 2017; Astratenkova and Rogozkin, 2019).

The role of HDACs in muscle metabolism has been extensively studied over the years. Early evidence revealed that Class IIa HDACs have a main role in regulating myofiber identity in physiological conditions. Indeed, Class IIa HDACs associate with MEF2 transcription factors ultimately repressing their transcriptional activity thereby regulating the metabolism of oxidative, slow-twitch myofibers (Wu et al., 2000; Potthoff et al., 2007). In particular, HDAC5 directly binds to the promoter region of *GLUT4*, repressing the transcription of this gene. This ultimately leads to the shift of muscle fibers toward oxidative metabolism promoting their glucose uptake (McGee et al., 2008).

The Class I members HDAC1 and HDAC2 are essential to skeletal muscle metabolism and homeostasis in physiological conditions. The deletion of HDAC1 and HDAC2 in skeletal muscle induces a shift toward a more oxidative metabolism and higher energy expenditure, caused by defects in the autophagy flux (Moresi et al., 2012). More recently, an important role in muscle energy metabolism was demonstrated for HDAC3 by means of its muscle-specific deletion: this causes an insulin resistance associated with higher endurance and fatigue resistance, due to a decrease in the uptake of glucose and an increase in the consumption of lipids (Hong et al., 2017; Song et al., 2019). The involvement of Class I HDACs in skeletal muscle metabolism was further demonstrated through the pharmacological inhibition of this class (Galmozzi et al., 2013), validating previous findings obtained through genetic approaches.

Being nicotinamide adenine dinucleotide (NAD+)-dependent enzymes, Sirtuins control gene transcription in response to changes in nutrient availability and energy demand. Indeed, an energy deficit increases the cellular NAD^+^/NADH ratio, thus activating Sirtuins which promote mitochondrial biogenesis and fatty acid oxidation in skeletal muscle, by deacetylating both histone and non-histone proteins (Houtkooper et al., 2012), as a response to the low energy condition (Ryall, 2012). It is worth noting that the deletion of SIRT1 in mice has modest effects on skeletal muscle metabolism (Menzies et al., 2013), implying a biological redundancy of this adaptative response.

In addition to regulating metabolic homeostasis in the whole body, by controlling insulin sensitivity and glucose tolerance (Bhaskara, 2018), the Class IV member HDAC11 has been recently reported to play a crucial role in skeletal muscle metabolism and function (Hurtado et al., 2021). HDAC11 localizes in muscle mitochondria and its deletion increases mitochondrial content, causing a glycolytic-to-oxidative muscle fiber switch (Hurtado et al., 2021).

Metabolic stresses, such as exercise, obesity, or a high fat diet (HFD), induce skeletal muscle adaptations through HDACs. Exercise modulates skeletal muscle metabolism *via* epigenetic reprogramming, partially through the phosphorylation-dependent nuclear export of Class II HDACs (McGee and Hargreaves, 2011). In addition, exercise also affects Sirtuin activity, as shown by the fact that a number of histone and non-histone Sirtuin targets are deacetylated during exercise (Cantó et al., 2009). Furthermore, obesity and HFD induce epigenetic reprogramming in skeletal muscle, involving Class I and II HDACs (Berdeaux et al., 2007; Sun et al., 2011; Moresi et al., 2012), although, the underlying mechanisms are not yet fully known.

Given the crucial role of HDACs in the regulation of muscle metabolism in various pathophysiological conditions, it is not surprising that not only HDACs in skeletal muscle are targeted by soluble factors, but also that they regulate the production of myokines affecting other tissues. The role played by myokines and adipo-myokines is outlined in the next paragraph, in which we describe how these factors of muscle and non-muscle origin are able to reprogram the metabolic activity of several tissues, ultimately determining metabolic adaptations in the whole body.

## Myokines as Mediators of Metabolic Reprogramming

Skeletal muscle is an important source of secreted factors exerting autocrine, paracrine and endocrine effects (Di Felice et al., 2020; Florin et al., 2020). This skeletal muscle secretome varies in response to ever-changing metabolic demand, dependent on variable physiological and pathological conditions (Lecompte et al., 2017; Florin et al., 2020). Among the numerous circulating factors involved in the regulation of systemic metabolism we included in our overview those that are expressed in the skeletal muscle and also have a proved link to HDACs.

Myostatin was the first characterized skeletal muscle-derived circulating protein (McPherron et al., 1997), originally identified in mice as a regulator of muscle growth *via* a negative feedback. Further studies clarified that myostatin targets other tissues in addition to muscle. For instance, myostatin is involved in the crosstalk between skeletal muscle and adipose tissue, thereby modulating insulin sensitivity and fat accumulation, both in animals and humans (Argilés et al., 2012). Moreover, myostatin targets bone, affecting osteogenic differentiation and enhancing resorption, modulating overall bone mass (Hamrick et al., 2006, 2007). Follistatin is a myostatin-binding protein and an inhibitor of myostatin activity, capable of promoting muscle growth (Yakabe et al., 2020). In humans, following exercise circulating follistatin increases and targets several organs, regulating pancreatic cell function and survival and promoting insulin resistance in skeletal muscle and adipose tissue (Willis et al., 2019). In this regard, we have shown that the mechanical stretch of murine myotubes *per se* alters the amount of myokines produced by muscle cells *in vitro*, including follistatin (Baccam et al., 2019).

In the search for a consensus term to define peptides produced and released by skeletal muscle, the word “myokine” referring to Interleukin 6 (IL-6) was first introduced in 2003 (Pedersen et al., 2003). Ten years later, the term “adipo-myokine” was coined to indicate those myokines, such as IL-6, that are also expressed by adipose tissue (Raschke and Eckel, 2013). In humans, the level of circulating IL-6 is increased after exercise (Northoff and Berg, 1991), mainly due to its secretion by skeletal muscle (Steensberg et al., 2000). Studies conducted in mice and humans highlighted that in response to physical exercise IL-6 exerts a positive role in modulating muscle mass, insulin secretion and lipid metabolism (Rosendal et al., 2005; Serrano et al., 2008; Ellingsgaard et al., 2011; da Silva Vasconcelos and Fernanda Salla, 2018). In addition, IL-6 produces anti-inflammatory effects, inhibiting the expression of Tumor Necrosis Factor-α (TNFα) and Interleukin-1 (IL-1) (Pedersen et al., 2003). However, besides the above mentioned positive effects, other studies highlighted the pleiotropic functions of this factor and revealed the deleterious effect of IL-6 on muscle homeostasis, showing that it is also a promoter of muscle wasting (Moresi et al., 2019). Indeed, IL-6 overexpression induces muscle atrophy in transgenic mice (Tsujinaka et al., 1996) and perturbs redox homeostasis (Forcina et al., 2019). Consistently, while the acute induction of IL-6 promotes muscle growth, sustained and elevated levels of IL-6 have been associated with muscle wasting in several human catabolic conditions (Yudkin et al., 2000; Kern et al., 2001; Pradhan et al., 2001; Ershler and Keller, 2003). All of the above shows how important it is to consider the complexity of a system where the kinetics and the source tissue of a given factor significantly influence its effects. To this regard IL-6 is emblematic and all these aspects should be taken into account when discussing the possible clinical implications of adipo-myokines.

Metrnl and BAIBA are two myokines whose expression is regulated by the transcriptional factor peroxisome proliferator-activated receptor-gamma coactivator-1α (PGC-1α) (Boström et al., 2012; Rao et al., 2014) and induced upon exercise in murine and human skeletal muscle (Rao et al., 2014; Roberts et al., 2014). These myokines target many tissues, exerting numerous physiological functions, such as promoting neural and osteocyte differentiation, preserving pancreatic β-cell functions and browning white adipose tissue (WAT), in addition to regulating glucose uptake and energy expenditure in skeletal muscle (Zheng et al., 2016; Darvin et al., 2018; Tanianskii et al., 2019; Rabiee et al., 2020).

Among other myokines, irisin production (Maak et al., 2021) has been shown to be induced by exercise in mice and humans (Boström et al., 2012; Jedrychowski et al., 2015). Studies with gain- (Boström et al., 2012) and loss- (Xiong et al., 2019) of function approaches demonstrate the ability of irisin to mediate the positive effects of physical activity in mice. These effects were not confirmed in humans (Maak et al., 2021).

The role of irisin, or even its existence in humans (Albrecht et al., 2015), is still debated, mainly in relation to: (1) the accuracy and reliability of the methods used to detect circulating irisin levels (Albrecht et al., 2015; Jedrychowski et al., 2015; Pourteymour et al., 2017; Bretland et al., 2021); (2) the positive effects of irisin on target organs or tissues (Raschke et al., 2013; Elsen et al., 2014; Maak et al., 2021). The debate on irisin exemplifies how difficult it is to study the crosstalk between tissues.

Metabolic stresses, such as acute bouts of exercise, fasting and caloric restriction, induce the expression of ANGPTL4 in human skeletal muscle (Catoire et al., 2014), likely by increasing plasma free-fatty acid levels and activating the peroxisome proliferator-activated receptor-δ (PPAR-δ) (Staiger et al., 2009). ANGPTL4 stimulates lipolysis in WAT, determining a shift from fat storage to fat release, and triggers AMP-activated protein kinase (AMPK) in skeletal muscle, thereby mediating an increase in its mitochondrial oxidative capacity and ATP production (Norheim et al., 2014; Chang et al., 2018; Li et al., 2020). It should be noted that besides skeletal muscle, adipose tissue and the liver express higher levels of ANGPTL4 following exercise, likely contributing even more than skeletal muscle to ANGPTL4 exercise-mediated effects (Norheim et al., 2014). Interestingly, human studies showed that ANGPTL4 mRNA in skeletal muscle is lower in men than in women at basal conditions, probably due to differences in the utilization of glucose and fat mass in the two genders (Barja-Fernandez et al., 2018; Li et al., 2020). Moreover, a positive correlation between muscle ANGPTL4, obesity and glucose metabolism has been reported: indeed, ANGPTL4 muscle and serum levels are significantly higher in obese patients with an abnormal glucose tolerance (Barja-Fernandez et al., 2018; Li et al., 2020).

Fibroblast growth factor 21 targets several organs including: (1) the liver, where it activates important metabolic responses, such as the induction of hepatic fatty acid oxidation, ketogenesis, and gluconeogenesis; (2) the pancreas, where it is necessary to maintain the digestive and endocrine functions of this organ; (3) WAT, where it promotes insulin sensitivity, glucose uptake, fatty acid storage, and oxidative capacity (Cuevas-Ramos et al., 2019; Martínez-Garza et al., 2019; Keinicke et al., 2020; Watanabe et al., 2020; Xie and Leung, 2020). Several cellular stress-signals, such as autophagy impairment, mitochondrial dysfunction and ER stress, induce FGF21 release from murine skeletal muscle (Kim and Lee, 2014; Pereira et al., 2017; Oost et al., 2019). All these induce FGF21 expression *via* the activation of transcription factor 4 (ATF4) (Keipert et al., 2014; Miyake et al., 2016). In addition, both mammalian target of rapamycin (mTOR) complex 1 (mTORC1) and mTORC2 have been reported to induce FGF21 expression in murine skeletal muscle (Guridi et al., 2015; Pereira et al., 2017). Furthermore, the secretion of FGF21 from human skeletal muscle is strongly induced upon exercise or in pathophysiological conditions including in mitochondrial myopathies and aging, suggesting the involvement of muscle-derived FGF21 in both healthy and pathological conditions (Hanks et al., 2015; Tanimura et al., 2016; Villarroya et al., 2018; Tezze et al., 2019). Despite its beneficial effect on body metabolism, the therapeutic role of FGF21 to the advantage of human health is still under debate, due to its paradoxical effects on different tissues. Indeed, FGF21 has negative effects on bone mass and mineral density both in mice and humans (Wei et al., 2012; Fazeli et al., 2015). Moreover, the long-term increase of circulating FGF21, triggered in skeletal muscle by stress conditions, contributes to systemic inflammation, precocious senescence, and premature death in mice (Tezze et al., 2017). Additional studies are required to clarify the specific role of FGF21 secreted from other organs and its effect on the whole body under physiological and pathological conditions.

Data available on rodents and humans highlight a rapid increase of IL-15 in circulation in response to exercise, improving whole body insulin sensitivity and decreasing adiposity (Carbó et al., 2001; Nadeau and Aguer, 2019). The data regarding IL-15 expression and release from skeletal muscle upon exercise in humans are controversial, probably due to the use of different exercise protocols in different studies or to differences in the subjects analyzed (Nielsen et al., 2007). In rats, autocrine IL-15 release induces a shift toward an oxidative skeletal muscle phenotype through the induction of PPAR-d and PGC-1α (Carbó et al., 2001; Busquets et al., 2006; Pistilli and Quinn, 2013), contributing to the beneficial adaptations to exercise of the metabolism (Hennigar et al., 2017). IL-15 also inhibits muscle protein degradation (Pistilli et al., 2007), however, since its levels decline progressively with aging, this contributes to age-related sarcopenia (Quinn, 2008; Quinn et al., 2010). In addition, IL-15 directly targets adipose tissue modulating the WAT mass and lipid metabolism probably because it decreases lipoprotein lipase (LPL) and leptin activities (Alvarez et al., 2002). A positive effect of a greater amount of circulating IL-15 on bone mineral content has been reported, likely due to the ability of IL-15 to interfere with TNF-α signaling (Quinn et al., 2009).

The expression of myonectin in murine skeletal muscle is regulated by the availability of nutrients and by exercise (Seldin et al., 2012; Laurens et al., 2020). In addition, patients with type 2 diabetes mellitus or those affected by obesity exhibit higher levels of circulating myonectin compared to healthy individuals (Li et al., 2018). Circulating myonectin has been shown to decrease the levels of circulating non-esterified fatty acid, through the promotion of fatty acid uptake both in hepatocytes and in adipocytes (Seldin et al., 2012). Moreover, the exercise-induced myonectin release from skeletal muscle protects cardiac myocytes from apoptosis, leading to a reduction of acute myocardial ischemia-reperfusion injury in mice (Otaka et al., 2017, 2018). Taken together, these data underline the role of myonectin in regulating systemic fatty acid metabolism and in mediating a cross-talk between muscle and other metabolic compartments (Seldin et al., 2012), suggesting possible therapeutic applications.

Brain-derived neurotrophic factor is a member of the neurotrophic factor family, whose expression is increased in human and rat skeletal muscles in response to muscle contraction (Dupont-Versteegden et al., 2004; Matthews et al., 2009). In human skeletal muscle, BDNF activates AMPK and promotes lipid oxidation following exercise (Matthews et al., 2009). In addition to skeletal muscle, exercise greatly increases the expression and release of BDNF from the brain, in both humans and mice (Liu and Nusslock, 2018), which, most likely, accounts for most of the BDNF-mediated effects, since the brain is the major source of this soluble factor.

The aforementioned studies taken together indicate that major metabolic adaptations to physiological or pathological alterations are regulated by soluble factors, mediating a complex crosstalk involving a network of tissues. Many of these circulating factors involve changes in HDAC expression or activation in skeletal muscle. The next paragraph focuses on this aspect.

## Myokines Affect Histone Deacetylase Expression and Activity

Myokines and other soluble factors control tissue homeostasis through a variety of signaling pathways including the regulation of HDAC expression or activity (Table 1). For instance, a correlation between the exercise-induced hormone irisin, known to regulate energy metabolism and to protect against numerous diseases, and HDAC4 has already been proposed. In rodents irisin protects the cardiomyocytes of rats exposed to hypoxic stress (Zhao et al., 2016) and the myocardium of type II diabetic db/db mice (Zhao et al., 2016; Wang et al., 2020). This protective role of irisin correlates with the degradation of HDAC4 (Zhao et al., 2016; Wang et al., 2020), which causes cell death and mitochondrial dysfunction in these cells. These findings highlight the principle, in rodents at least, that a systemic factor, namely irisin, produces its effects partially involving epigenetic mechanisms. Since the relevance of irisin-mediated effects in humans is still debated, more studies are needed to clarify whether the protective role of irisin on muscle in our species exists, and whether it involves epigenetic mechanisms.

**TABLE 1:** Circulating factors affect histone deacetylase (HDAC) expression or activity in various tissues.

Species	Circulating factors	Target tissue	Downstream effects	Link with HDAC	References
Rat	BDNF	Neurons	Activation of MEF2	HDAC5 nuclear export	Finsterwald et al., 2013
Mouse	Fgf21	Skeletal muscle	Myoblast differentiation, improved aerobic metabolism	SIRT1 activation	Liu et al., 2017
Mouse	Fgf21	Adipocytes	Increased mitochondrial oxidative capacity	SIRT1 activation	Chau et al., 2010
Mouse	Fgf21	Hepatocytes, germ cells, cardiomyocytes	Protection from oxidative stress, apoptosis, and fibrosis	SIRT1 activation	Zhu et al., 2014; Jiang et al., 2015; Zhang et al., 2016; Abd Elwahab et al., 2017; Wang et al., 2017; Li et al., 2019
Mouse	IL-15	Skeletal muscle	Improved oxidative metabolism	SIRT1 activation	Quinn et al., 2011, 2013, 2014
Rodents	Irisin	Cardiomyocytes	Protection from death	HDAC4 degradation	Zhao et al., 2016; Wang et al., 2020
Mouse	Leptin	Skeletal muscle	Acetylation of PGC-1α	SIRT1 activation	García-Carrizo et al., 2016
Mouse	Leptin	Macrophages	Reduction of inflammation	HDAC4 nuclear import	Luan et al., 2014
Mouse	Metrnl	Skeletal muscle	Glucose uptake	HDAC5 nuclear export	Lee et al., 2020

*The table represents an overview of the correlation between circulating factors and changes in the HDAC expression or activation (Link with HDAC) in a given tissue (Target tissue). The final output (Downstream effects) is also reported, as well as the species where the observations were made and the corresponding references.*

**FIGURE 1:**
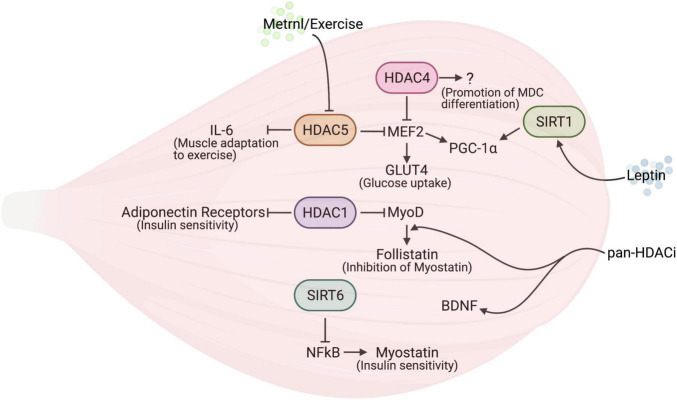
The interplay between histone deacetylases (HDACs) and myokines in skeletal muscle. The reciprocal regulation of HDACs and myokines modulates skeletal muscle metabolism.

The secretion of Metrnl by skeletal muscle, increased during muscle contractions, improves glucose tolerance by inducing glucose uptake *via* the AMPK-dependent phosphorylation of HDAC5, a transcriptional repressor of *Glut4* in mice (Lee et al., 2020): the phosphorylated HDAC5 interacts with 14-3-3 proteins, is sequestrated in the cytoplasm, and results in the activation of *Glut4* transcription (Figure 1). Glucose uptake by the musculature represents a paradigmatic behavior, which usually occurs in response to the classic insulin signaling. The interesting aspect of the alternative Metrnl signaling stems from the fact that the upregulation of the *Glut4* gene involves an energy-sensing factor, AMPK, as well as an epigenetic factor, HDAC5. In addition, the delivery of recombinant Metrnl improves glucose tolerance in mice with HFD-induced obesity or type 2 diabetes (Lee et al., 2020), suggesting the potential use of this myokine as a therapeutic tool to treat metabolic disorders.

Fibroblast growth factor 21 activates a SIRT1-AMPK-PGC1α pathway in skeletal muscle, thereby promoting myoblast differentiation and aerobic metabolism, and is also possibly involved in starvation-induced muscle atrophy (Liu et al., 2017). In adipocytes the same axis has been proven to be responsible for increasing mitochondrial oxidative capacity (Chau et al., 2010). Additionally, the protective role of the FGF21-SIRT1 axis in different pathological conditions has been reported in multiple cell types, suggesting that this pathway may be a potential target for therapeutic approaches. Indeed, FGF21 activates SIRT1 in hepatocytes, thereby ameliorating multiple metabolic parameters in alcoholic fatty liver disease (Zhu et al., 2014) or cafeteria-diet induced steatohepatitis (Abd Elwahab et al., 2017). In addition, FGF21 also activates SIRT1 in germ cells reducing their oxidative stress and apoptosis in diabetes (Jiang et al., 2015). A similar TGF21-induced SIRT1 activation in cardiomyocytes has a protective effect on type 1 diabetes-induced inflammation and fibrosis in the heart (Zhang et al., 2016), on doxorubicin-induced cardiomyopathy (Wang et al., 2017) as well as on angiotensin-II induced cardiomyocyte apoptosis and cardiac hypertrophy (Li et al., 2019).

Interleukin 15, a myokine which contributes to the benefits of physical exercise on muscle metabolism, is responsible for the post-exercise induction of SIRT1 in muscle, which, in turn, mediates many of the metabolic effects of physical exercise (Quinn et al., 2013, 2014). IL-15 Tg mice also exhibit an induction of the SIRT1 protein and changes in oxidative metabolism, which may be involved in their phenotype, i.e., the resistance to diet-induced obesity and higher insulin-sensitivity (Quinn et al., 2011).

Differently from FGF21 and IL-15, ANGPTL4 represses *Sirt1* expression in hepatocytes, thus mediating the inflammatory response in acute lung injury (Guo et al., 2015).

Leptin has been shown to target multiple tissues, including skeletal muscle. Evidence of a direct effect of leptin on muscle cells comes from *in vitro* experiments showing that, in murine myotubes, leptin induces the expression of genes involved in metabolism (including PGC-1α, uncoupling protein 3, muscle carnitine palmitoyltransferase 1) in addition to that of myokines such as IL-6 and IL-15 (Nozhenko et al., 2015). Interestingly, leptin affects SIRT1-mediated PGC-1α deacetylation (García-Carrizo et al., 2016; Figure 1), even though contradictory data have been reported. This is probably due to dissimilar leptin and nutrient concentrations in the culture media, as well as to different timing of the analyses. *In vivo*, leptin receptor deficiency in skeletal muscle leads to muscle atrophy and compromised primary myoblast proliferation and differentiation (Arounleut et al., 2013), indicating a possible role for this adipokine in skeletal muscle homeostasis. Whether or not leptin influences adult skeletal muscle *via* modulating HDACs has not been investigated yet. However, an initial link between leptin and HDACs has been reported: indeed, the administration of leptin in conjunction with a HFD induces HDAC4 dephosphorylation and its nuclear import in macrophages in white adipose tissue, thereby reducing inflammation (Luan et al., 2014). The molecular mechanism described for leptin-induced HDAC dephosphorylation consists in a catecholamine-dependent increase in cAMP, triggered by leptin, which inhibits salt-inducible kinases. This ultimately promotes HDAC4 dephosphorylation; the dephosphorylated HDAC4 then shuttles to the nucleus where it inhibits NF-κB transcriptional activity on pro-inflammatory genes (Luan et al., 2014).

Although experimental evidence in skeletal muscle cells is still lacking, it is interesting to note that BDNF activates the salt-inducible kinase 1 (SIK1), a member of the AMPK family, in rat neurons, leading to the phosphorylation-dependent nuclear extrusion of HDAC5 and the consequent MEF2-dependent gene activation (Finsterwald et al., 2013). It would be important to investigate this signaling in skeletal muscles as well, since it may underly the beneficial effects of BDNF on muscle metabolism.

So far, we have reported that myokines control the metabolic shifts occurring in multiple tissues in response to physiological or pathological stress conditions, and that, at least in muscle tissues, myokines are capable of inducing epigenetic responses through HDACs. Conversely, HDACs influence the release of several factors from skeletal muscle, as outlined next.

## Histone Deacetylases Affect Myokine Synthesis

Histone deacetylases regulate the expression of chemokines, cytokines and adipo-myokines, or that of their receptors, under physiological and pathological conditions in several tissues (Gatla et al., 2019). In particular, recent studies highlighted this role of HDACs in skeletal muscle—and in other cells to a lesser extent (Table 2). A paramount example of this is the fact that HDAC4 regulates factors secreted by skeletal muscle upon injury, thus regulating muscle-derived cell (MDC) myogenic potential and muscle regeneration (Renzini et al., 2018). Indeed, the deletion of HDAC4 in skeletal muscle hampers skeletal muscle regeneration, even though MDCs from HDAC4 KO mice differentiate well when cultured *in vitro*, i.e., isolated from their muscle niche. Conversely, sera from injured HDAC4 KO mice negatively affect MDC differentiation, which proves that HDAC4 affects the release of soluble factors from skeletal muscle upon injury, which, in turn, are important for muscle regeneration (Renzini et al., 2018; Figure 1). The characterization of the secretome of HDAC4 KO will clarify this new important function of HDAC4 in skeletal muscle and provide new insights for a potential therapeutic application to muscle regeneration.

**TABLE 2:** HDACs regulate the production of myokines.

HDAC	Circulating factor	Target tissue	Downstream effects	References
HDACs	Follistatin	Skeletal muscle, FAPs	Promotion of myogenesis	Iezzi et al., 2004; Minetti et al., 2006; Mozzetta et al., 2013
HDACs	BDNF	Skeletal muscle, motor neurons	Improvement of the myofiber maturation and of the motor unit morphology and function	Avila et al., 2007
HDACs	ANGPTL4	Adipose tissue	Improved lipolysis	Zhang et al., 2021
HDAC1	Adiponectin	Skeletal muscle	Protection from HFD-induced obesity and insulin resistance; stimulation of mitochondrial function	Jian et al., 2016
HDAC2, HDAC3	Fgf21	Glia cells, adipocytes	Regulation of the outgrowth of extending processes and of the fatty acid utilization	Li et al., 2012; Leng et al., 2016
HDAC4	Unknown	Skeletal muscle	Promotion of muscle cell differentiation	Renzini et al., 2018
HDAC5	IL-6	Skeletal muscle	Muscle adaptation to exercise	Klymenko et al., 2020
SIRT1	Fgf21	Hepatocytes, heart	Improved energy expenditure	Li et al., 2014; Furukawa et al., 2020
SIRT6	Myostatin	Skeletal muscle	Promotion of myogenesis	Samant et al., 2017

*The table shows how the activity of members of different HDAC classes affect the production of myokines (Circulating Factors) ultimately eliciting multiple responses (Downstream effects). For each of these findings the corresponding reference is cited.*

Myostatin is a member of the transforming growth factor-beta (TGF-beta) superfamily, expressed and released by skeletal muscle and that negatively influences muscle growth (McPherron et al., 1997). Myostatin expression is controlled by SIRT6, which regulates the binding of NF-kB to the myostatin promoter (Samant et al., 2017; Figure 1). Indeed, SIRT6 KO mice display muscle degeneration and atrophy, in addition to reduced fat and bone density. Conversely, the SIRT6 overexpression counteracts cytokine-induced myostatin expression in muscle cells, promoting myogenesis. Therefore, increasing SIRT6 activity or expression has been proposed as a potential therapeutic tool to counteract many disease states characterized by muscle cachexia, thanks to the ability of SIRT6 to influence myostatin expression and possibly act on multiple tissues.

Follistatin is a secreted protein able to bind with and neutralize the actions of many members of the TGF-beta family of proteins (Patel, 1998). General Pan-HDAC inhibitors (HDACi), such as valproic acid (VPA) or Trichostatin-A (TSA), have been shown to increase the expression of follistatin in muscle cells, through the binding of MyoD to the follistatin promoter, a process which is fostered by local hyperacetylation (Figure 1); in turn, the release of the soluble factor follistatin counteracts myostatin activity, enhancing myogenesis (Iezzi et al., 2004). Further studies clarified that pan-HDACi improve muscle regeneration in dystrophy by increasing the expression of follistatin in both muscle stem cells and fibro-adipogenic progenitors (Minetti et al., 2006; Mozzetta et al., 2013)—a finding that led scientists to suggest the use of HDACi in clinical trials for the treatment of Duchenne Muscular Dystrophy. Although pan-HDACi increase the expression of follistatin in cancer cachexia as well, this is not sufficient to prevent muscle wasting in this disease state (Bonetto et al., 2009). Also, pan-HDACi have been shown to increase BDNF expression in the skeletal muscles and spinal cord in a murine model of Spinal Muscular Atrophy (SMA) (Avila et al., 2007; Figure 1). Since BDNF is an important contraction-induced secreted factor for the neurogenesis, growth, and survival of neurons (Murawska-Ciałowicz et al., 2021), its increase may explain the beneficial effects of HDACi on SMA progression.

Interleukin 6 was recognized in the past as a principal mediator of muscle adaptation to exercise (Ostrowski et al., 1998). Muscle contractions induce an increase of IL-6 gene expression that leads to a release of IL-6 protein in the circulation (Reihmane and Dela, 2013). An epigenetic modulation of IL-6 expression driven by HDAC5 has been recently reported in exercised skeletal muscle. Genetic murine models helped clarify the metabolic changes, including that of glucose metabolism, regulated by the HDAC5 activity on the IL-6 promoter (Klymenko et al., 2020): in resting conditions, HDAC5 negatively modulates IL-6 transcription by removing histone H3 acetylation at lysine 9 (AcH3K9), while upon exercise, HDAC5 is exported to the cytoplasm and results in an increased expression of GLUT4, thus prompting skeletal muscle cells to uptake glucose in response to insulin (Klymenko et al., 2020; Figure 1).

Histone deacetylases regulate ANGPTL4 expression in different cell types. ANGPTL4 is a secreted glycoprotein which inhibits the activity of lipoprotein lipases, thereby increasing circulating triglyceride levels (Fernández-Hernando and Suárez, 2020). HDACi sodium butyrate activates lipolysis in pigs, in part by downregulating *ANGPTL4* gene expression in the adipose tissue (Zhang et al., 2021). In human cells, HDAC3 mediates a NCOR-dependent transcriptional repression of the *ANGPTL4* gene, as shown by using a renal cancer cell line, while additional investigations are needed to unveil the potential role of HDAC in regulating *ANGPTL4* expression in skeletal muscle.

Different HDACs exert opposite effects on the expression of *Fgf21*, an important regulator of carbohydrate and lipid metabolism, in different cell types. HDAC2 and HDAC3 inhibit *Fgf21* expression in glia cells, thus regulating the outgrowth of their processes (Leng et al., 2016), and in adipocytes, affecting fatty acid utilization and ketogenesis (Li et al., 2012). On the contrary, SIRT1 induces *Fgf21* expression in hepatocytes, preventing fat-induced liver steatosis while promoting white adipose tissue browning and higher energy expenditure (Li et al., 2014). SIRT1 also induces *Fgf21* expression in the heart, improving energy metabolism and increasing the contractile efficiency of pressure-overloaded hearts (Furukawa et al., 2020).

A correlation between SIRT1 expression and irisin serum levels has been reported in type 2 diabetic patients and mice (Safarpour et al., 2020; Jiang et al., 2021), suggesting the involvement of SIRT1 in PGC-1α-mediated irisin release. The treatment of obese type 2 diabetes patients with vitamin D also induces higher serum levels of SIRT1 and irisin, further pointing to the SIRT1-mediated release of irisin (Safarpour et al., 2020). The SIRT1 activator resveratrol, alone or in combination with all-trans retinoic acid (ATRA), significantly induces the expression of irisin in skeletal muscle cells (RongXia et al., 2017; Abedi-Taleb et al., 2019). Consistently, metformin, another compound activating SIRT1, increases irisin expression and its release from skeletal muscle in diabetic mice (Yang et al., 2015). In spite of the limited evidence to date, it seems likely that the positive effects of SIRT1 activation on body metabolism (Feige et al., 2008) are at least in part due to its ability to increase the levels of circulating irisin.

In skeletal muscle HDACs regulate signaling in response to adiponectin. This is an adipokine involved in controlling glucose levels and fatty acid breakdown (Yamauchi et al., 2001), which, interestingly, also plays a role in skeletal muscle (Fruebis, 2001). Here HDAC1 represses the transcription of the genes encoding adiponectin receptors and uncoupling protein 2 and 3. Since the latter are important mitochondrial proteins involved in heat generation and energy expenditure, the inhibition of HDAC1 contributes to the beneficial effects of the pan-HDACi sodium butyrate on HFD-induced obesity in mice (Jian et al., 2016; Figure 1).

Based on the substantial experimental evidence above, it is apparent that myokines, cytokines and adipokines control the homeostatic and metabolic responses of tissues at organismal level, while their dysregulation is responsible for many diseases. Since HDACs affect the synthesis and ultimately the release of these factors in various organs or, vice-versa, modulate the organ response to endocrine factors through the regulation of their receptors, HDACs are very promising pharmaceutical targets. However, the first attempts to interfere with the activity of HDACs by means of HDACi must be further supported by additional investigations on the specific role of each HDAC family member. This approach alone will provide a solid ground for the development of HDACi-based therapies for human diseases. The studies on HDACi performed so far are summarized below.

## Clinical Implications of the Use of Histone Deacetylase Inhibitors

Several preclinical studies highlighted the positive effects of HDACi in regulating the whole-body metabolism in various diabetic or insulin-resistance rodent models, thus providing the rationale for the inclusion of HDACi in clinical trials targeting metabolic disorders. For instance, pan-HDACi sodium butyrate and TSA, which improve insulin sensitivity and energy expenditure in HFD-fed mice (Gao et al., 2009; Li et al., 2012; Lin et al., 2012; Henagan et al., 2015; Xu et al., 2020), increase the liver expression of FGF21 and its serum concentration (Li et al., 2012). Consequently, FGF21 mediates the increase in fatty acid utilization and ketogenesis, in response to butyrate treatment, causing resistance to HFD. An added value offered by this study is the fact that it represents an additional proof of principle that many HDACi effects on body metabolism may be achieved through the regulation of soluble factors, such as FGF21 itself. Butyrate improves glucose and pyruvate tolerance in aged mice, reducing fat and preserving skeletal muscle mass (Walsh et al., 2015)—a result that suggests the use of HDACi to counter aging. Butyrate also increases mitochondrial biogenesis and oxygen consumption in the skeletal muscle of aged mice; notably, the use of this HDACi does not affect the HDAC4-myogenin axis, which is significant in neurogenic muscle atrophy (Moresi et al., 2010). In addition, another pan-HDACi, Scriptaid, modulates adaptative metabolic responses to exercise (Gaur et al., 2016). Considering the beneficial effects of exercise on aging, all these findings further suggest new future applications for HDACi in the context of sarcopenia (Pasyukova and Vaiserman, 2017).

In spite of the numerous positive effects that HDACi exert on metabolism control, additional considerations are needed concerning the use of pan-HDACi in clinical trials for metabolic disorders. The use of general HDACi is usually discouraged, since their use is normally associated with numerous side effects and since they target a broad range of proteins. This makes drawing any biological conclusion on the action of specific HDACs difficult. Indeed, contradictory data were reported on the effectiveness of specific Classes of HDACi in ameliorating insulin resistance in obese mice (Sharma and Taliyan, 2016), probably due to the different concentrations used in the various studies or to the fact that Class II enzymatic activity depends on Class I HDACs, which can bias the effects of HDACi (Fischle et al., 2002).

Thus, further investigations should be carried out, both at the pre-clinical and clinical level, before proposing the unspecific pharmacological inhibition of this important enzyme family. The two major avenues of research in this field should be aimed at defining: (1) expression and activity of the different members of the HDAC family, under pathophysiological conditions, and (2) the functions of each single HDAC member in different tissues. Only by undertaking this approach will it be possible to design enzyme- and tissue-specific pharmacological treatments.

## Conclusion

Myokines are mediators of the crosstalk between skeletal muscle and other tissues of pivotal importance for body metabolism and they support the central role of skeletal muscle as a metabolic hub. HDACs not only are regulators of muscle metabolism, they also affect other tissues, including liver and fat tissues, by controlling myokine production. The effects of this production can be muscle -centered, as is the case of myostatin and related factors, or have a broader action, such as in the case of pro- and anti-inflammatory cytokines. In turn, HDAC activity is regulated in response to the humoral milieu, a fact which suggests how important epigenetic control is to fine-tune the genetic response of tissues to hormones or other circulating factors. Given the broad and pleiotropic effects of HDACs, these may be targets in the treatment of metabolic and systemic pathological conditions.

## Author Contributions

VM conceived the manuscript. AR and MD’O generated the figure. DC supervised the content of the text. All authors contributed to the writing.

## Conflict of Interest

The authors declare that the research was conducted in the absence of any commercial or financial relationships that could be construed as a potential conflict of interest.

## Publisher’s Note

All claims expressed in this article are solely those of the authors and do not necessarily represent those of their affiliated organizations, or those of the publisher, the editors and the reviewers. Any product that may be evaluated in this article, or claim that may be made by its manufacturer, is not guaranteed or endorsed by the publisher.

## Abbreviations

AcH3K9: Acetyl-Histone H3 (Lys9)
AMPK: AMP-activated protein kinase
ANGPTL4: Angiopoietin-like protein 4
ATF4: Activate transcription factor 4
AP-1: Activator protein 1
BAIBA: β-aminoisobutyric acid
BDNF: Brain-derived neurotrophic factor
cAMP: Cyclic adenosine monophosphate
ER: Endoplasmic Reticulum
FGF-21: Fibroblast growth factor 21
GLUT4: Glucose transporter type 4
HAT: Histone Acetyltransferases
HDAC: Histone Deacetylases
HDACi: HDAC inhibitors
HFD: High fat diet
IL-1: Interleukin 1
IL-6: Interleukin 6
IL-15: Interleukin 15
IKK: Nuclear factor-kB Kinase
JNK: c-JunN-terminal kinase
LPL: Lipoprotein lipase
MDC: Muscle-derived cells
MEF2: Myocyte enhancer factor-2
Metrnl: Meteorin-like
mTOR: Mammalian target of rapamycin
MyoD: Myoblast determination protein 1
NAD: Nicotinamide adenine dinucleotide
NFkB: Nuclear factor kappa-light-chain-enhancer of activated B cells
TNFα: Tumor Necrosis Factor-α
TSA: Trichostatin-A
PGC-1α: Peroxisome proliferator-activated receptor-gamma coactivator-1α
PPAR-δ: Peroxisome proliferator-activated receptor-δ
SIK1: Salt-inducible kinase 1
SIRT: Sirtuin
STAT3: Signal Transducer And Activator Of Transcription 3
VPA: Valproic acid
WAT: White adipose tissue.

## References

[B1] Abd Elwahab A. H. Ramadan B. K. Schaalan M. F. Tolba A. M. (2017). A novel role of SIRT1/FGF-21 in taurine protection against cafeteria diet-induced steatohepatitis in rats. *Cell. Physiol. Biochem.* 43 644–659. 10.1159/000480649 28942443

[B2] Abedi-Taleb E. Vahabi Z. Sekhavati-Moghadam E. Khedmat L. Jazayeri S. Saboor-Yaraghi A. A. (2019). Upregulation of FNDC5 gene expression in C2C12 cells after single and combined treatments of resveratrol and ATRA. *Lipids Health Dis.* 18:181. 10.1186/S12944-019-1128-Y 31640715 PMC6806552

[B3] Ahima R. S. Park H. K. (2015). Connecting myokines and metabolism. *Endocrinol. Metab.* 30 235–245. 10.3803/EnM.2015.30.3.235 26248861 PMC4595346

[B4] Albrecht E. Norheim F. Thiede B. Holen T. Ohashi T. Schering L. (2015). Irisin – a myth rather than an exercise-inducible myokine. *Sci. Rep.* 5:8889. 10.1038/srep08889 25749243 PMC4352853

[B5] Alvarez B. Carbó N. López-Soriano J. Drivdahl R. H. Busquets S. López-Soriano F. J. (2002). Effects of interleukin-15 (IL-15) on adipose tissue mass in rodent obesity models: evidence for direct IL-15 action on adipose tissue. *Biochim. Biophys. Acta Gen. Subj.* 1570 33–37. 10.1016/S0304-4165(02)00148-4 11960686

[B6] Argilés J. M. Orpí M. Busquets S. López-Soriano F. J. (2012). Myostatin: more than just a regulator of muscle mass. *Drug Discov. Today* 17 702–709. 10.1016/j.drudis.2012.02.001 22342983

[B7] Arounleut P. Bowser M. Upadhyay S. Shi X. M. Fulzele S. Johnson M. H. (2013). Erratum: absence of functional leptin receptor isoforms in the POUND (Leprdb/lb) mouse is associated with muscle atrophy and altered myoblast proliferation and differentiation. *PLoS One* 8:8. 10.1371/annotation/3a7d6e24-137c-4603-93ca-879bec7fab80 PMC3743798 23967295

[B8] Astratenkova I. V. Rogozkin V. A. (2019). The role of acetylation/deacetylation of histones and transcription factors in regulating metabolism in skeletal muscles. *Neurosci. Behav. Physiol.* 49 281–288. 10.1007/s11055-019-00730-2

[B9] Avila A. M. Burnett B. G. Taye A. A. Gabanella F. Knight M. A. Hartenstein P. (2007). Trichostatin A increases SMN expression and survival in a mouse model of spinal muscular atrophy. *J. Clin. Invest.* 117 659–671. 10.1172/JCI29562 17318264 PMC1797603

[B10] Baccam A. Benoni-Sviercovich A. Rocchi M. Moresi V. Seelaender M. Li Z. (2019). The mechanical stimulation of myotubes counteracts the effects of tumor-derived factors through the modulation of the activin/follistatin ratio. *Front. Physiol.* 10:401. 10.3389/fphys.2019.00401 31068826 PMC6491697

[B11] Bannister A. J. Kouzarides T. (2011). Regulation of chromatin by histone modifications. *Cell Res.* 21 381–395. 10.1038/cr.2011.22 21321607 PMC3193420

[B12] Barja-Fernandez S. Moreno-Navarrete J. M. Folgueira C. Xifra G. Sabater M. Castelao C. (2018). Plasma ANGPTL-4 is associated with obesity and glucose tolerance: cross-sectional and longitudinal findings. *Mol. Nutr. Food Res.* 62:e1800060. 10.1002/mnfr.201800060 29536615

[B13] Baskin K. K. Winders B. R. Olson E. N. (2015). Muscle as a “mediator” of systemic metabolism. *Cell Metab.* 21 237–248. 10.1016/j.cmet.2014.12.021 25651178 PMC4398026

[B14] Berardi E. Madaro L. Lozanoska-Ochser B. Adamo S. Thorrez L. Bouche M. (2021). A pound of flesh: what cachexia is and what it is not. *Diagnostics* 11:116. 10.3390/diagnostics11010116 33445790 PMC7828214

[B15] Berdeaux R. Goebel N. Banaszynski L. Takemori H. Wandless T. Shelton G. D. (2007). SIK1 is a class II HDAC kinase that promotes survival of skeletal myocytes. *Nat. Med.* 13 597–603. 10.1038/nm1573 17468767

[B16] Berezin A. E. Berezin A. A. Lichtenauer M. (2021). Myokines and heart failure: challenging role in adverse cardiac remodeling, myopathy, and clinical outcomes. *Dis. Markers* 2021:6644631. 10.1155/2021/6644631 33520013 PMC7819753

[B17] Bhaskara S. (2018). Histone deacetylase 11 as a key regulator of metabolism and obesity. *EBioMedicine* 35 27–28. 10.1016/j.ebiom.2018.08.008 30126820 PMC6154867

[B18] Bianchi M. Renzini A. Adamo S. Moresi V. (2017). Coordinated actions of microRNAs with other epigenetic factors regulate skeletal muscle development and adaptation. *Int. J. Mol. Sci.* 18:840. 10.3390/ijms18040840 28420141 PMC5412424

[B19] Bonetto A. Penna F. Minero V. Reffo P. Bonelli G. Baccino F. (2009). Deacetylase inhibitors modulate the myostatin/follistatin axis without improving cachexia in tumor-bearing mice. *Curr. Cancer Drug Targets* 9 608–616. 10.2174/156800909789057015 19508174

[B20] Boström P. Wu J. Jedrychowski M. P. Korde A. Ye L. Lo J. C. (2012). A PGC1-α-dependent myokine that drives brown-fat-like development of white fat and thermogenesis. *Nature* 481 463–468. 10.1038/nature10777 22237023 PMC3522098

[B21] Bretland K. A. Lin L. Bretland K. M. Smith M. A. Fleming S. M. Dengler-Crish C. M. (2021). Irisin treatment lowers levels of phosphorylated tau in the hippocampus of pre-symptomatic female but not male htau mice. *Neuropathol. Appl. Neurobiol.* 47 967–978. 10.1111/NAN.12711 33768561 PMC9292848

[B22] Busquets S. Figueras M. Almendro V. López-Soriano F. J. Argilés J. M. (2006). Interleukin-15 increases glucose uptake in skeletal muscle An antidiabetogenic effect of the cytokine. *Biochim. Biophys. Acta Gen. Subj.* 1760 1613–1617. 10.1016/j.bbagen.2006.09.001 17056184

[B23] Cantó C. Gerhart-Hines Z. Feige J. N. Lagouge M. Noriega L. Milne J. C. (2009). AMPK regulates energy expenditure by modulating NAD+ metabolism and SIRT1 activity. *Nature* 458 1056–1060. 10.1038/nature07813 19262508 PMC3616311

[B24] Carbó N. López-Soriano J. Costelli P. Alvarez B. Busquets S. Baccino F. M. (2001). Interleukin-15 mediates reciprocal regulation of adipose and muscle mass: a potential role in body weight control. *Biochim. Biophys. Acta Gen. Subj.* 1526 17–24. 10.1016/S0304-4165(00)00188-4 11287118

[B25] Catoire M. Alex S. Paraskevopulos N. Mattijssen F. Evers-Van Gogh I. Schaart G. (2014). Fatty acid-inducible ANGPTL4 governs lipid metabolic response to exercise. *Proc. Natl. Acad. Sci. U. S. A.* 111 E1043–52. 10.1073/pnas.1400889111 24591600 PMC3964070

[B26] Chang H. Kwon O. Shin M.-S. Kang G. M. Leem Y. H. Lee C. H. (2018). Role of Angptl4/Fiaf in exercise-induced skeletal muscle AMPK activation. *J. Appl. Physiol.* 125 715–722. 10.1152/japplphysiol.00984.2016 29952246

[B27] Chau M. D. Gao J. Yang Q. Wu Z. Gromada J. (2010). Fibroblast growth factor 21 regulates energy metabolism by activating the AMPK–SIRT1–PGC-1α pathway. *Proc. Natl. Acad. Sci. U. S. A.* 107 12553–12558. 10.1073/PNAS.1006962107 20616029 PMC2906565

[B28] Chen W. Zhang X. Birsoy K. Roeder R. G. (2010). A muscle-specific knockout implicates nuclear receptor coactivator MED1 in the regulation of glucose and energy metabolism. *Proc. Natl. Acad. Sci. U. S. A.* 107 10196–10201. 10.1073/pnas.1005626107 20479251 PMC2890439

[B29] Correia J. C. Ferreira D. M. Ruas J. L. (2015). Intercellular: local and systemic actions of skeletal muscle PGC-1s. *Trends Endocrinol. Metab.* 26 305–314. 10.1016/j.tem.2015.03.010 25934582

[B30] Cuevas-Ramos D. Mehta R. Aguilar-Salinas C. A. (2019). Fibroblast growth factor 21 and browning of white adipose tissue. *Front. Physiol.* 10:37. 10.3389/fphys.2019.00037 30804796 PMC6370737

[B31] da Silva Vasconcelos E. Fernanda Salla R. (2018). Role of interleukin-6 and interleukin-15 in exercise. *MOJ Immunol.* 6:00185. 10.15406/MOJI.2018.06.00185

[B32] Daou N. Hassani M. Matos E. De Castro G. S. Costa R. G. F. Seelaender M. (2020). Displaced myonuclei in cancer cachexia suggest altered innervation. *Int. J. Mol. Sci.* 21:1092. 10.3390/ijms21031092 32041358 PMC7038037

[B33] Darvin P. Toor S. M. Sasidharan Nair V. Elkord E. (2018). Immune checkpoint inhibitors: recent progress and potential biomarkers. *Exp. Mol. Med.* 50:165. 10.1038/s12276-018-0191-1 30546008 PMC6292890

[B34] de Lima E. A. Teixeira A. A. de S. Biondo L. A. Diniz T. A. Silveira L. S. (2020). Exercise reduces the resumption of tumor growth and proteolytic pathways in the skeletal muscle of mice following chemotherapy. *Cancers (Basel)* 12:3466. 10.3390/cancers12113466 33233839 PMC7699885

[B35] Di Felice V. Coletti D. Seelaender M. (2020). Editorial: myokines, adipokines, cytokines in muscle pathophysiology. *Front. Physiol.* 11:592856. 10.3389/fphys.2020.592856 33192608 PMC7645054

[B36] Dupont-Versteegden E. E. Houlé J. D. Dennis R. A. Zhang J. Knox M. Wagoner G. (2004). Exercise-induced gene expression in soleus muscle is dependent on time after spinal cord injury in rats. *Muscle Nerve* 29 73–81. 10.1002/MUS.10511 14694501

[B37] Ellingsgaard H. Hauselmann I. Schuler B. Habib A. M. Baggio L. L. Meier D. T. (2011). Interleukin-6 enhances insulin secretion by increasing glucagon-like peptide-1 secretion from L cells and alpha cells. *Nat. Med.* 17 1481–1489. 10.1038/nm.2513 22037645 PMC4286294

[B38] Elsen M. Raschke S. Eckel J. (2014). Browning of white fat: does irisin play a role in humans? *J. Endocrinol.* 222 R25–R38. 10.1530/JOE-14-0189 24781257

[B39] Ershler W. B. Keller E. T. (2003). Age-associated increased interleukin-6 gene expression, late-life diseases, and frailty. *Annu. Rev. Med.* 51 245–270. 10.1146/ANNUREV.MED.51.1.245 10774463

[B40] Evans W. S. Blumenthal J. B. Heilman J. M. Ryan A. S. Prior S. J. (2021). Effects of exercise training with weight loss on skeletal muscle expression of angiogenic factors in overweight and obese older men. *J. Appl. Physiol. (1985)* 131 56–63. 10.1152/JAPPLPHYSIOL.00084.2021 34013746 PMC8325618

[B41] Fazeli P. K. Faje A. T. Cross E. J. Lee H. Rosen C. J. Bouxsein M. L. (2015). Serum FGF-21 levels are associated with worsened radial trabecular bone microarchitecture and decreased radial bone strength in women with anorexia nervosa. *Bone* 77 6–11. 10.1016/j.bone.2015.04.001 25868802 PMC4447546

[B42] Feige J. N. Lagouge M. Canto C. Strehle A. Houten S. M. Milne J. C. (2008). Specific SIRT1 activation mimics low energy levels and protects against diet-induced metabolic disorders by enhancing fat oxidation. *Cell Metab.* 8 347–358. 10.1016/J.CMET.2008.08.017 19046567

[B43] Fernández-Hernando C. Suárez Y. (2020). ANGPTL4: a multifunctional protein involved in metabolism and vascular homeostasis. *Curr. Opin. Hematol.* 27 206–213. 10.1097/MOH.0000000000000580 32205586 PMC9013473

[B44] Finsterwald C. Carrard A. Martin J.-L. (2013). Role of salt-inducible kinase 1 in the activation of MEF2-dependent transcription by BDNF. *PLoS One* 8:e54545. 10.1371/journal.pone.0054545 23349925 PMC3551851

[B45] Fischle W. Dequiedt F. Hendzel M. J. Guenther M. G. Lazar M. A. Voelter W. (2002). Enzymatic activity associated with class II HDACs is dependent on a multiprotein complex containing HDAC3 and SMRT/N-CoR. *Mol. Cell* 9 45–57. 10.1016/S1097-2765(01)00429-4 11804585

[B46] Florin A. Lambert C. Sanchez C. Zappia J. Durieux N. Tieppo A. M. (2020). The secretome of skeletal muscle cells: a systematic review. *Osteoarthr. Cartil. Open* 2:100019. 10.1016/j.ocarto.2019.100019 PMC9718214 36474563

[B47] Forcina L. Miano C. Scicchitano B. M. Rizzuto E. Berardinelli M. G. De Benedetti F. (2019). Increased circulating levels of interleukin-6 affect the redox balance in skeletal muscle. *Oxid. Med. Cell. Longev.* 2019 1–13. 10.1155/2019/3018584 31827671 PMC6881749

[B48] Fruebis J. (2001). Proteolytic cleavage product of 30-kDa adipocyte complement-related protein increases fatty acid oxidation in muscle and causes weight loss in mice. *Proc. Natl. Acad. Sci. U. S. A.* 98 2005–2010. 10.1073/pnas.041591798 11172066 PMC29372

[B49] Frye R. A. (1999). Characterization of five human cDNAs with homology to the yeast SIR2 gene: sir2-like proteins (Sirtuins) metabolize NAD and may have protein ADP-ribosyltransferase activity. *Biochem. Biophys. Res. Commun.* 260 273–279. 10.1006/bbrc.1999.0897 10381378

[B50] Furukawa N. Koitabashi N. Matsui H. Sunaga H. Umbarawan Y. Syamsunarno M. R. A. A. (2020). DPP-4 inhibitor induces FGF21 expression *via* sirtuin 1 signaling and improves myocardial energy metabolism. *Heart Vessels* 361 136–146. 10.1007/S00380-020-01711-Z 33073318 PMC7788045

[B51] Galmozzi A. Mitro N. Ferrari A. Gers E. Gilardi F. Godio C. (2013). Inhibition of class I histone deacetylases unveils a mitochondrial signature and enhances oxidative metabolism in skeletal muscle and adipose tissue. *Diabetes* 62 732–742. 10.2337/db12-0548 23069623 PMC3581211

[B52] Gannon J. Doran P. Kirwan A. Ohlendieck K. (2009). Drastic increase of myosin light chain MLC-2 in senescent skeletal muscle indicates fast-to-slow fibre transition in sarcopenia of old age. *Eur. J. Cell Biol.* 88 685–700. 10.1016/j.ejcb.2009.06.004 19616867

[B53] Gao Z. Yin J. Zhang J. Ward R. E. Martin R. J. Lefevre M. (2009). Butyrate improves insulin sensitivity and increases energy expenditure in mice. *Diabetes* 58 1509–1517. 10.2337/db08-1637 19366864 PMC2699871

[B54] García-Carrizo F. Nozhenko Y. Palou A. Rodríguez A. M. (2016). Leptin effect on acetylation and phosphorylation of Pgc1α in muscle cells associated With Ampk and Akt activation in high-glucose medium. *J. Cell. Physiol.* 231 641–649. 10.1002/jcp.25109 26218179

[B55] Gatla H. R. Muniraj N. Thevkar P. Yavvari S. Sukhavasi S. Makena M. R. (2019). Regulation of chemokines and cytokines by histone deacetylases and an update on histone decetylase inhibitors in human diseases. *Int. J. Mol. Sci.* 20:1110. 10.3390/ijms20051110 30841513 PMC6429312

[B56] Gaur V. Connor T. Sanigorski A. Martin S. D. Bruce C. R. Henstridge D. C. (2016). Disruption of the class IIa HDAC corepressor complex increases energy expenditure and lipid oxidation. *Cell Rep.* 16 2802–2810. 10.1016/j.celrep.2016.08.005 27626651

[B57] Glozak M. A. Sengupta N. Zhang X. Seto E. (2005). Acetylation and deacetylation of non-histone proteins. *Gene* 363 15–23. 10.1016/J.GENE.2005.09.010 16289629

[B58] Guo L. Li S. Zhao Y. Qian P. Ji F. Qian L. (2015). Silencing angiopoietin-like protein 4 (ANGPTL4) protects against lipopolysaccharide-induced acute lung injury *via* regulating SIRT1/NF-kB pathway. *J. Cell. Physiol.* 230 2390–2402. 10.1002/JCP.24969 25727991

[B59] Guridi M. Tintignac L. A. Lin S. Kupr B. Castets P. Rüegg M. A. (2015). Activation of mTORC1 in skeletal muscle regulates whole-body metabolism through FGF21. *Sci. Signal.* 8:ra113. 10.1126/scisignal.aab3715 26554817

[B60] Haberland M. Montgomery R. L. Olson E. N. (2009). The many roles of histone deacetylases in development and physiology: implications for disease and therapy. *Nat. Rev. Genet.* 10 32–42. 10.1038/nrg2485 19065135 PMC3215088

[B61] Hamrick M. W. Samaddar T. Pennington C. McCormick J. (2006). Increased muscle mass with myostatin deficiency improves gains in bone strength with exercise. *J. Bone Miner. Res.* 21 477–483. 10.1359/JBMR.051203 16491296

[B62] Hamrick M. W. Shi X. Zhang W. Pennington C. Thakore H. Haque M. (2007). Loss of myostatin (GDF8) function increases osteogenic differentiation of bone marrow-derived mesenchymal stem cells but the osteogenic effect is ablated with unloading. *Bone* 40 1544–1553. 10.1016/j.bone.2007.02.012 17383950 PMC2001954

[B63] Hanks L. J. Gutiérrez O. M. Bamman M. M. Ashraf A. McCormick K. L. Casazza K. (2015). Circulating levels of fibroblast growth factor-21 increase with age independently of body composition indices among healthy individuals. *J. Clin. Transl. Endocrinol.* 2 77–82. 10.1016/j.jcte.2015.02.001 26042208 PMC4450097

[B64] Henagan T. M. Stefanska B. Fang Z. Navard A. M. Ye J. Lenard N. R. (2015). Sodium butyrate epigenetically modulates high-fat diet-induced skeletal muscle mitochondrial adaptation, obesity and insulin resistance through nucleosome positioning. *Br. J. Pharmacol.* 172 2782–2798. 10.1111/bph.13058 25559882 PMC4439875

[B65] Hennigar S. R. McClung J. P. Pasiakos S. M. (2017). Nutritional interventions and the IL-6 response to exercise. *FASEB J.* 31 3719–3728. 10.1096/FJ.201700080R 28507168

[B66] Hong S. Zhou W. Fang B. Lu W. Loro E. Damle M. (2017). Dissociation of muscle insulin sensitivity from exercise endurance in mice by HDAC3 depletion. *Nat. Med.* 23 223–234. 10.1038/nm.4245 27991918 PMC5540654

[B67] Houtkooper R. H. Pirinen E. Auwerx J. (2012). Sirtuins as regulators of metabolism and healthspan. *Nat. Rev. Mol. Cell Biol.* 13 225–238. 10.1038/nrm3293 22395773 PMC4872805

[B68] Howlett K. F. McGee S. L. (2016). Epigenetic regulation of skeletal muscle metabolism. *Clin. Sci.* 130 1051–1063. 10.1042/CS20160115 27215678

[B69] Hurtado E. Núñez-Álvarez Y. Muñoz M. Gutiérrez-Caballero C. Casas J. Pendás A. M. (2021). HDAC11 is a novel regulator of fatty acid oxidative metabolism in skeletal muscle. *FEBS J.* 288 902–919. 10.1111/febs.15456 32563202

[B70] Hutchinson K. A. Mohammad S. Garneau L. McInnis K. Aguer C. Adamo K. B. (2019). Examination of the myokine response in pregnant and non-pregnant women following an acute bout of moderate-intensity walking. *Front. Physiol.* 10:1188. 10.3389/fphys.2019.01188 31649549 PMC6795697

[B71] Hyndman K. A. Knepper M. A. (2017). Dynamic regulation of lysine acetylation: the balance between acetyltransferase and deacetylase activities. *Am. J. Physiol. Ren. Physiol.* 313 F842–F846. 10.1152/ajprenal.00313.2017 28701313 PMC5668585

[B72] Iezzi S. Di Padova M. Serra C. Caretti G. Simone C. Maklan E. (2004). Deacetylase inhibitors increase muscle cell size by promoting myoblast recruitment and fusion through induction of follistatin. *Dev. Cell* 6 673–684. 10.1016/S1534-5807(04)00107-8 15130492

[B73] Jacques M. Hiam D. Craig J. Barrès R. Eynon N. Voisin S. (2019). Epigenetic changes in healthy human skeletal muscle following exercise– a systematic review. *Epigenetics* 14 633–648. 10.1080/15592294.2019.1614416 31046576 PMC6557592

[B74] Jedrychowski M. P. Wrann C. D. Paulo J. A. Gerber K. K. Szpyt J. Robinson M. M. (2015). Detection and quantitation of circulating human irisin by tandem mass spectrometry. *Cell Metab.* 22 734–740. 10.1016/J.CMET.2015.08.001 26278051 PMC4802359

[B75] Jian H. Yimin J. Shifng P. Longfei J. Huifang L. Zhenqiang H. (2016). Butyrate alleviates high fat diet-induced obesity through activation of adiponectin-mediated pathway and stimulation of mitochondrial function in the skeletal muscle of mice. *Oncotarget* 7 56071–56082. 10.18632/oncotarget.11267 27528227 PMC5302897

[B76] Jiang S. Piao L. Ma E. B. Ha H. Huh J. Y. (2021). Associations of circulating irisin with FNDC5 expression in fat and muscle in type 1 and type 2 diabetic mice. *Biomolecules* 11 1–14. 10.3390/BIOM11020322 33672565 PMC7924053

[B77] Jiang X. Chen J. Zhang C. Zhang Z. Tan Y. Feng W. (2015). The protective effect of FGF21 on diabetes-induced male germ cell apoptosis is associated with up-regulated testicular AKT and AMPK/Sirt1/PGC-1α signaling. *Endocrinology* 156 1156–1170. 10.1210/EN.2014-1619 25560828 PMC6285187

[B78] Keinicke H. Sun G. Mentzel C. M. J. Fredholm M. John L. M. Andersen B. (2020). FGF21 regulates hepatic metabolic pathways to improve steatosis and inflammation. *Endocr. Connect.* 9 755–768. 10.1530/EC-20-0152 32688339 PMC7424338

[B79] Keipert S. Ost M. Johann K. Imber F. Jastroch M. van Schothorst E. M. (2014). Skeletal muscle mitochondrial uncoupling drives endocrine cross-talk through the induction of FGF21 as a myokine. *Am. J. Physiol. Endocrinol. Metab.* 306 E469–E482. 10.1152/ajpendo.00330.2013 24347058

[B80] Kern P. A. Ranganathan S. Li C. Wood L. Ranganathan G. (2001). Adipose tissue tumor necrosis factor and interleukin-6 expression in human obesity and insulin resistance. *Am. J. Physiol. Endocrinol. Metab.* 280 E745–E751. 10.1152/AJPENDO.2001.280.5.E745 11287357

[B81] Kim K. H. Lee M. S. (2014). FGF21 as a stress hormone: the roles of FGF21 in stress adaptation and the treatment of metabolic disease. *Diabetes Metab. J.* 38 245–251. 10.4093/dmj.2014.38.4.245 25215270 PMC4160577

[B82] Klymenko O. Brecklinghaus T. Dille M. Springer C. de Wendt C. Altenhofen D. (2020). Histone deacetylase 5 regulates interleukin 6 secretion and insulin action in skeletal muscle. *Mol. Metab.* 42:101062. 10.1016/j.molmet.2020.101062 32771698 PMC7481569

[B83] Kurgan N. Noaman N. Pergande M. R. Cologna S. M. Coorssen J. R. Klentrou P. (2019). Changes to the human serum proteome in response to high intensity interval exercise: a sequential top-down proteomic analysis. *Front. Physiol.* 10:362. 10.3389/fphys.2019.00362 31001142 PMC6454028

[B84] Laurens C. Bergouignan A. Moro C. (2020). Exercise-released myokines in the control of energy metabolism. *Front. Physiol.* 11:91. 10.3389/fphys.2020.00091 32116795 PMC7031345

[B85] Leal L. G. Lopes M. A. Batista M. L. (2018). Physical exercise-induced myokines and muscle-adipose tissue crosstalk: a review of current knowledge and the implications for health and metabolic diseases. *Front. Physiol.* 9:1307. 10.3389/fphys.2018.01307 30319436 PMC6166321

[B86] Lecompte S. Abou-Samra M. Boursereau R. Noel L. Brichard S. M. (2017). Skeletal muscle secretome in Duchenne muscular dystrophy: a pivotal anti-inflammatory role of adiponectin. *Cell. Mol. Life Sci.* 7413 2487–2501. 10.1007/S00018-017-2465-5 28188344 PMC5487898

[B87] Lee J. O. Byun W. S. Kang M. J. Han J. A. Moon J. Shin M. J. (2020). The myokine meteorin-like (metrnl) improves glucose tolerance in both skeletal muscle cells and mice by targeting AMPKα2. *FEBS J.* 287 2087–2104. 10.1111/febs.15301 32196931 PMC7383816

[B88] Leng Y. Wang J. Wang Z. Liao H.-M. Wei M. Leeds P. (2016). Valproic acid and other HDAC inhibitors upregulate FGF21 gene expression and promote process elongation in glia by inhibiting HDAC2 and 3. *Int. J. Neuropsychopharmacol.* 19:pyw035. 10.1093/IJNP/PYW035 27207921 PMC5006201

[B89] Li G. Zhang H. Ryan A. S. (2020). Skeletal muscle angiopoietin-like protein 4 and glucose metabolism in older adults after exercise and weight loss. *Metabolites* 10:354. 10.3390/metabo10090354 32878157 PMC7570075

[B90] Li H. Gao Z. Zhang J. Ye X. Xu A. Ye J. (2012). Sodium butyrate stimulates expression of fibroblast growth factor 21 in liver by inhibition of histone deacetylase 3. *Diabetes* 61 797–806. 10.2337/db11-0846 22338096 PMC3314370

[B91] Li K. Liao X. Wang K. Mi Q. Zhang T. Jia Y. (2018). Myonectin predicts the development of type 2 diabetes. *J. Clin. Endocrinol. Metab.* 103 139–147. 10.1210/jc.2017-01604 29161407

[B92] Li S. Zhu Z. Xue M. Yi X. Liang J. Niu C. (2019). Fibroblast growth factor 21 protects the heart from angiotensin II-induced cardiac hypertrophy and dysfunction *via* SIRT1. *Biochim. Biophys. Acta Mol. Basis Dis.* 1865 1241–1252. 10.1016/J.BBADIS.2019.01.019 30677512

[B93] Li Y. Seto E. (2016). HDACs and HDAC inhibitors in cancer development and therapy. *Cold Spring Harb. Perspect. Med.* 6:a026831. 10.1101/cshperspect.a026831 27599530 PMC5046688

[B94] Li Y. Wong K. Giles A. Jiang J. Lee J. W. Adams A. C. (2014). Hepatic SIRT1 attenuates hepatic steatosis and controls energy balance in mice by inducing fibroblast growth factor 21. *Gastroenterology* 146 539–49.e7. 10.1053/J.GASTRO.2013.10.059 24184811 PMC4228483

[B95] Lin H. V. Frassetto A. Kowalik E. J. Jr. Nawrocki A. R. Lu M. M. Kosinski J. R. (2012). Butyrate and propionate protect against diet-induced obesity and regulate gut hormones *via* free fatty acid receptor 3-independent mechanisms. *PLoS One* 7:e35240. 10.1371/journal.pone.0035240 22506074 PMC3323649

[B96] Liu J. J. Foo J. P. Liu S. Lim S. C. (2015). The role of fibroblast growth factor 21 in diabetes and its complications: a review from clinical perspective. *Diabetes Res. Clin. Pract.* 108 382–389. 10.1016/J.DIABRES.2015.02.032 25796513

[B97] Liu P. Z. Nusslock R. (2018). Exercise-mediated neurogenesis in the hippocampus *via* BDNF. *Front. Neurosci.* 12:52. 10.3389/FNINS.2018.00052 29467613 PMC5808288

[B98] Liu X. Wang Y. Hou L. Xiong Y. Zhao S. (2017). Fibroblast Growth Factor 21 (FGF21) promotes formation of aerobic myofibers *via* the FGF21-SIRT1-AMPK-PGC1α Pathway. *J. Cell. Physiol.* 232 1893–1906. 10.1002/JCP.25735 27966786

[B99] Luan B. Goodarzi M. O. Phillips N. G. Guo X. Chen Y. D. I. Yao J. (2014). Leptin-mediated increases in catecholamine signaling reduce adipose tissue inflammation *via* activation of macrophage HDAC4. *Cell Metab.* 19 1058–1065. 10.1016/j.cmet.2014.03.024 24768298 PMC4207085

[B100] Maak S. Norheim F. Drevon C. A. Erickson H. P. (2021). Progress and Challenges in the Biology of FNDC5 and Irisin. *Endocr. Rev.* 42 436–456. 10.1210/ENDREV/BNAB003 33493316 PMC8284618

[B101] Martínez-Garza Ú. Torres-Oteros D. Yarritu-Gallego A. Marrero P. F. Haro D. Relat J. (2019). Fibroblast growth factor 21 and the adaptive response to nutritional challenges. *Int. J. Mol. Sci.* 20:4692. 10.3390/ijms20194692 31546675 PMC6801670

[B102] Matthews V. B. Åström M. B. Chan M. H. S. Bruce C. R. Krabbe K. S. Prelovsek O. (2009). Brain-derived neurotrophic factor is produced by skeletal muscle cells in response to contraction and enhances fat oxidation *via* activation of AMP-activated protein kinase. *Diabetologia* 52 1409–1418. 10.1007/s00125-009-1364-1 19387610

[B103] McGee S. L. Hargreaves M. (2011). Histone modifications and exercise adaptations. *J. Appl. Physiol.* 110 258–263. 10.1152/japplphysiol.00979.2010 21030677

[B104] McGee S. L. Van Denderen B. J. W. Howlett K. F. Mollica J. Schertzer J. D. Kemp B. E. (2008). AMP-activated protein kinase regulates GLUT4 transcription by phosphorylating histone deacetylase 5. *Diabetes* 57 860–867. 10.2337/db07-0843 18184930

[B105] McPherron A. C. Lawler A. M. Lee S. J. (1997). Regulation of skeletal muscle mass in mice by a new TGF-beta superfamily member. *Nature* 387 83–90. 10.1038/387083A0 9139826

[B106] Menzies K. J. Singh K. Saleem A. Hood D. A. (2013). Sirtuin 1-mediated effects of exercise and resveratrol on mitochondrial biogenesis. *J. Biol. Chem.* 288 6968–6979. 10.1074/jbc.M112.431155 23329826 PMC3591607

[B107] Michishita E. Park J. Y. Burneskis J. M. Barrett J. C. Horikawa I. (2005). Evolutionarily conserved and nonconserved cellular localizations and functions of human SIRT proteins. *Mol. Biol. Cell* 16 4623–4635. 10.1091/mbc.E05-01-0033 16079181 PMC1237069

[B108] Minetti G. C. Colussi C. Adami R. Serra C. Mozzetta C. Parente V. (2006). Functional and morphological recovery of dystrophic muscles in mice treated with deacetylase inhibitors. *Nat. Med.* 12 1147–1150. 10.1038/nm1479 16980968

[B109] Miyake M. Nomura A. Ogura A. Takehana K. Kitahara Y. Takahara K. (2016). Skeletal muscle-specific eukaryotic translation initiation factor 2α phosphorylation controls amino acid metabolism and fibroblast growth factor 21-mediated non-cell-autonomous energy metabolism. *FASEB J.* 30 798–812. 10.1096/fj.15-275990 26487695 PMC4945323

[B110] Moresi V. Adamo S. Berghella L. (2019). The JAK/STAT pathway in skeletal muscle pathophysiology. *Front. Physiol.* 10:500. 10.3389/fphys.2019.00500 31114509 PMC6502894

[B111] Moresi V. Carrer M. Grueter C. E. Rifki O. F. Shelton J. M. Richardson J. A. (2012). Histone deacetylases 1 and 2 regulate autophagy flux and skeletal muscle homeostasis in mice. *Proc. Natl. Acad. Sci. U. S. A.* 109 1649–1654. 10.1073/pnas.1121159109 22307625 PMC3277131

[B112] Moresi V. Marroncelli N. Pigna E. Adamo S. (2016). “Epigenetics of muscle disorders,” in *Medical Epigenetics*, ed. Tollefsbol T. (Amsterdam: Elsevier Inc), 315–333. 10.1016/B978-0-12-803239-8.00018-1

[B113] Moresi V. Williams A. H. Meadows E. Flynn J. M. Potthoff M. J. McAnally J. (2010). Myogenin and Class II HDACs control neurogenic muscle atrophy by inducing E3 ubiquitin ligases. *Cell* 143 35–45. 10.1016/j.cell.2010.09.004 20887891 PMC2982779

[B114] Mozzetta C. Consalvi S. Saccone V. Tierney M. Diamantini A. Mitchell K. J. (2013). Fibroadipogenic progenitors mediate the ability of HDAC inhibitors to promote regeneration in dystrophic muscles of young, but not old Mdx mice. *EMBO Mol. Med.* 5 626–639. 10.1002/emmm.201202096 23505062 PMC3628105

[B115] Murawska-Ciałowicz E. Wiatr M. Ciałowicz M. de Assis G. G. Borowicz W. Rocha-Rodrigues S. (2021). BDNF impact on biological markers of depression-role of physical exercise and training. *Int. J. Environ. Res. Public Health* 18:7553. 10.3390/IJERPH18147553 34300001 PMC8307197

[B116] Nadeau L. Aguer C. (2019). Interleukin-15 as a myokine: mechanistic insight into its effect on skeletal muscle metabolism. *Appl. Physiol. Nutr. Metab.* 44 229–238. 10.1139/apnm-2018-0022 30189147

[B117] Nielsen A. R. Mounier R. Plomgaard P. Mortensen O. H. Penkowa M. Speerschneider T. (2007). Expression of interleukin-15 in human skeletal muscle – effect of exercise and muscle fibre type composition. *J. Physiol.* 584 305–12. 10.1113/JPHYSIOL.2007.139618 17690139 PMC2277063

[B118] Norheim F. Hjorth M. Langleite T. M. Lee S. Holen T. Bindesbøll C. (2014). Regulation of angiopoietin-like protein 4 production during and after exercise. *Physiol. Rep.* 2:e12109. 10.14814/phy2.12109 25138789 PMC4246580

[B119] Northoff H. Berg A. (1991). Immunologic mediators as parameters of the reaction to strenuous exercise. *Int. J. Sports Med.* 12 S9–S15. 10.1055/S-2007-1024743 1910016

[B120] Nozhenko Y. Rodríguez A. M. Palou A. (2015). Leptin rapidly induces the expression of metabolic and myokine genes in C2C12 muscle cells to regulate nutrient partition and oxidation. *Cell. Physiol. Biochem.* 35 92–103. 10.1159/000369678 25547246

[B121] Oost L. J. Kustermann M. Armani A. Blaauw B. Romanello V. (2019). Fibroblast growth factor 21 controls mitophagy and muscle mass. *J. Cachexia Sarcopenia Muscle* 10 630–642. 10.1002/jcsm.12409 30895728 PMC6596457

[B122] Ostrowski K. Rohde T. Zacho M. Asp S. Pedersen B. K. (1998). Evidence that interleukin-6 is produced in human skeletal muscle during prolonged running. *J. Physiol.* 508 949–953. 10.1111/j.1469-7793.1998.949bp.x 9518745 PMC2230908

[B123] Otaka N. Shibata R. Ohashi K. Murohara T. Ouchi N. (2017). P5393Myonectin/CTRP15 protects against myocardial ischemia-reperfusion injury. *Eur. Heart J.* 38:5393. 10.1093/eurheartj/ehx493.P5393

[B124] Otaka N. Shibata R. Ohashi K. Uemura Y. Kambara T. Enomoto T. (2018). Myonectin is an exercise-induced Myokine that protects the heart from ischemia-reperfusion injury. *Circ. Res.* 123 1326–1338. 10.1161/CIRCRESAHA.118.313777 30566056

[B125] Park S. Y. Kim J. S. (2020). A short guide to histone deacetylases including recent progress on class II enzymes. *Exp. Mol. Med.* 52 204–212. 10.1038/s12276-020-0382-4 32071378 PMC7062823

[B126] Pasyukova E. G. Vaiserman A. M. (2017). HDAC inhibitors: a new promising drug class in anti-aging research. *Mech. Ageing Dev.* 166 6–15. 10.1016/j.mad.2017.08.008 28843433

[B127] Patel K. (1998). Follistatin. *Int. J. Biochem. Cell Biol.* 30 1087–1093. 10.1016/S1357-2725(98)00064-8 9785474

[B128] Pedersen B. K. Steensberg A. Fischer C. Keller C. Keller P. Plomgaard P. (2003). Searching for the exercise factor: is IL-6 a candidate? J Muscle Res Cell Motil. 24 113–119. 10.1023/A:1026070911202 14609022

[B129] Pereira R. O. Tadinada S. M. Zasadny F. M. Oliveira K. J. Pires K. M. P. Olvera A. (2017). OPA1 deficiency promotes secretion of FGF21 from muscle that prevents obesity and insulin resistance. *EMBO J.* 36 2126–2145. 10.15252/embj.201696179 28607005 PMC5510002

[B130] Peserico A. Simone C. (2011). Physical and functional HAT/HDAC interplay regulates protein acetylation balance. *J. Biomed. Biotechnol.* 2011 1–10. 10.1155/2011/371832 21151613 PMC2997516

[B131] Piccirillo R. (2019). Exercise-induced myokines with therapeutic potential for muscle wasting. *Front. Physiol.* 10:287. 10.3389/fphys.2019.00287 30984014 PMC6449478

[B132] Pigna E. Simonazzi E. Sanna K. Bernadzki K. M. Proszynski T. Heil C. (2019). Histone deacetylase 4 protects from denervation and skeletal muscle atrophy in a murine model of amyotrophic lateral sclerosis. *EBioMedicine* 40 717–732. 10.1016/j.ebiom.2019.01.038 30713114 PMC6414308

[B133] Pin F. Bonewald L. F. Bonetto A. (2021). Role of myokines and osteokines in cancer cachexia. *Exp. Biol. Med. (Maywood)* 246 2118–2127. 10.1177/15353702211009213 33899538 PMC8524772

[B134] Pistilli E. E. Quinn L. S. (2013). From anabolic to oxidative: reconsidering the roles of IL-15 and IL-15Rα in skeletal muscle. *Exerc. Sport Sci. Rev.* 41 100–106. 10.1097/JES.0b013e318275d230 23072822 PMC5317349

[B135] Pistilli E. E. Siu P. M. Alway S. E. (2007). Interleukin-15 responses to aging and unloading-induced skeletal muscle atrophy. *Am. J. Physiol. Physiol.* 292 C1298–C1304. 10.1152/ajpcell.00496.2006 17135303

[B136] Potthoff M. J. Wu H. Arnold M. A. Shelton J. M. Backs J. McAnally J. (2007). Histone deacetylase degradation and MEF2 activation promote the formation of slow-twitch myofibers. *J. Clin. Invest.* 117 2459–2467. 10.1172/JCI31960 17786239 PMC1957540

[B137] Pourteymour S. Eckardt K. Holen T. Langleite T. Lee S. Jensen J. (2017). Global mRNA sequencing of human skeletal muscle: search for novel exercise-regulated myokines. *Mol. Metab.* 6 352–365. 10.1016/J.MOLMET.2017.01.007 28377874 PMC5369209

[B138] Pradhan A. D. Manson J. E. Rifai N. Buring J. E. Ridker P. M. (2001). C-Reactive protein, interleukin 6, and risk of developing type 2 diabetes mellitus. *JAMA* 286 327–334. 10.1001/JAMA.286.3.327 11466099

[B139] Quinn L. S. (2008). Interleukin-15: a muscle-derived cytokine regulating fat-to-lean body composition1,2. *J. Anim. Sci.* 86 E75–E83. 10.2527/jas.2007-0458 17709786

[B140] Quinn L. S. Anderson B. G. Conner J. D. Wolden-Hanson T. (2013). IL-15 overexpression promotes endurance, oxidative energy metabolism, and muscle PPARδ, SIRT1, PGC-1α, and PGC-1β expression in male mice. *Endocrinology* 154 232–245. 10.1210/EN.2012-1773 23161867 PMC3529369

[B141] Quinn L. S. Anderson B. G. Conner J. D. Pistilli E. E. Wolden-Hanson T. (2011). Overexpression of interleukin-15 in mice promotes resistance to diet-induced obesity, increased insulin sensitivity, and markers of oxidative skeletal muscle metabolism. *Int. J. Interf. Cytokine Mediat. Res.* 3 29–42. 10.2147/IJICMR.S19007 28943758 PMC5605924

[B142] Quinn L. S. Anderson B. G. Conner J. D. Wolden-Hanson T. Marcell T. J. (2014). IL-15 is required for postexercise induction of the pro-oxidative mediators PPARδ and SIRT1 in male mice. *Endocrinology* 155 143–155. 10.1210/EN.2013-1645 24169546 PMC5378429

[B143] Quinn L. S. Anderson B. G. Strait-Bodey L. Wolden-Hanson T. (2010). Serum and muscle interleukin-15 levels decrease in aging mice: correlation with declines in soluble interleukin-15 receptor alpha expression. *Exp. Gerontol.* 45 106–112. 10.1016/j.exger.2009.10.012 19854259 PMC2814937

[B144] Quinn L. S. Anderson B. G. Strait-Bodey L. Stroud A. M. Argués J. M. (2009). Oversecretion of interleukin-15 from skeletal muscle reduces adiposity. *Am. J. Physiol. Endocrinol. Metab.* 296 E191–202. 10.1152/ajpendo.90506.2008 19001550 PMC2636988

[B145] Rabiee F. Lachinani L. Ghaedi S. Nasr-Esfahani M. H. Megraw T. L. Ghaedi K. (2020). New insights into the cellular activities of Fndc5/Irisin and its signaling pathways. *Cell Biosci.* 10:51. 10.1186/s13578-020-00413-3 32257109 PMC7106581

[B146] Rao R. R. Long J. Z. White J. P. Svensson K. J. Lou J. Lokurkar I. (2014). Meteorin-like is a hormone that regulates immune-adipose interactions to increase beige fat thermogenesis. *Cell* 157 1279–1291. 10.1016/j.cell.2014.03.065 24906147 PMC4131287

[B147] Raschke S. Eckel J. (2013). Adipo-Myokines: two sides of the same coin–Mediators of inflammation and mediators of exercise. *Mediators Inflamm.* 2013:320724. 10.1155/2013/320724 23861558 PMC3686148

[B148] Raschke S. Elsen M. Gassenhuber H. Sommerfeld M. Schwahn U. Brockmann B. (2013). Evidence against a beneficial effect of irisin in humans. *PLoS One* 8:e73680. 10.1371/JOURNAL.PONE.0073680 24040023 PMC3770677

[B149] Reihmane D. Dela F. (2013). Interleukin-6: possible biological roles during exercise. *Eur. J. Sport Sci.* 14 242–250. 10.1080/17461391.2013.776640 24655147

[B150] Renzini A. Marroncelli N. Noviello C. Moresi V. Adamo S. (2018). HDAC4 regulates skeletal muscle regeneration *via* soluble factors. *Front. Physiol.* 9:1387. 10.3389/fphys.2018.01387 30319457 PMC6171007

[B151] Roberts L. D. Boström P. O’Sullivan J. F. Schinzel R. T. Lewis G. D. Dejam A. (2014). β-aminoisobutyric acid induces browning of white fat and hepatic β-oxidation and is inversely correlated with cardiometabolic risk factors. *Cell Metab.* 19 96–108. 10.1016/J.CMET.2013.12.003 24411942 PMC4017355

[B152] RongXia W. TaoYan Y. WanQiu Z. ShuJing L. JunWang T. Xue D. (2017). Effect of resveratrol on mRNA expression of chicken myokines. *Chin. J. Vet. Sci.* 37 364–368.

[B153] Rosendal L. Søgaard K. Kjær M. Sjøgaard G. Langberg H. Kristiansen J. (2005). Increase in interstitial interleukin-6 of human skeletal muscle with repetitive low-force exercise. *J. Appl. Physiol.* 98 477–481. 10.1152/JAPPLPHYSIOL.00130.2004 15448117

[B154] Ryall J. G. (2012). The role of sirtuins in the regulation of metabolic homeostasis in skeletal muscle. *Curr. Opin. Clin. Nutr. Metab. Care* 15 561–566. 10.1097/MCO.0b013e3283590914 23075935

[B155] Safarpour P. Daneshi-Maskooni M. Vafa M. Nourbakhsh M. Janani L. Maddah M. (2020). Vitamin D supplementation improves SIRT1, Irisin, and glucose indices in overweight or obese type 2 diabetic patients: a double-blind randomized placebo-controlled clinical trial. *BMC Fam. Pract.* 21:26. 10.1186/S12875-020-1096-3/TABLES/6 32033527 PMC7007689

[B156] Samant S. A. Kanwal A. Pillai V. B. Bao R. Gupta M. P. (2017). The histone deacetylase SIRT6 blocks myostatin expression and development of muscle atrophy. *Sci. Rep.* 7:11877. 10.1038/s41598-017-10838-5 28928419 PMC5605688

[B157] Schiaffino S. Reggiani C. (2011). Fiber types in mammalian skeletal muscles. *Physiol. Rev.* 91 1447–1531. 10.1152/physrev.00031.2010 22013216

[B158] Seldin M. M. Peterson J. M. Byerly M. S. Wei Z. Wong G. W. (2012). Myonectin (CTRP15), a novel myokine that links skeletal muscle to systemic lipid homeostasis. *J. Biol. Chem.* 287 11968–11980. 10.1074/jbc.M111.336834 22351773 PMC3320944

[B159] Serrano A. L. Baeza-Raja B. Perdiguero E. Jardí M. Muñoz-Cánoves P. (2008). Interleukin-6 is an essential regulator of satellite cell-mediated skeletal muscle hypertrophy. *Cell Metab.* 7 33–44. 10.1016/j.cmet.2007.11.011 18177723

[B160] Seto E. Yoshida M. (2014). Erasers of histone acetylation: the histone deacetylase enzymes. *Cold Spring Harb. Perspect. Biol.* 6:a018713. 10.1101/cshperspect.a018713 24691964 PMC3970420

[B161] Severinsen M. C. K. Pedersen B. K. (2020). Muscle–organ crosstalk: the emerging roles of myokines. *Endocr. Rev.* 41 594–609. 10.1210/ENDREV/BNAA016 32393961 PMC7288608

[B162] Sharma S. Taliyan R. (2016). Histone deacetylase inhibitors: future therapeutics for insulin resistance and type 2 diabetes. *Pharmacol. Res.* 113 320–326. 10.1016/j.phrs.2016.09.009 27620069

[B163] Sharples A. P. Stewart C. E. Seaborne R. A. (2016). Does skeletal muscle have an ‘epi’-memory? The role of epigenetics in nutritional programming, metabolic disease, aging and exercise. *Aging Cell* 15 603–616. 10.1111/acel.12486 27102569 PMC4933662

[B164] Simoneau J. Colberg S. R. Thaete F. L. Kelley D. E. (1995). Skeletal muscle glycolytic and oxidative enzyme capacities are determinants of insulin sensitivity and muscle composition in obese women. *FASEB J.* 9 273–278. 10.1096/fasebj.9.2.7781930 7781930

[B165] Song S. Wen Y. Tong H. Loro E. Gong Y. Liu J. (2019). The HDAC3 enzymatic activity regulates skeletal muscle fuel metabolism. *J. Mol. Cell Biol.* 11 133–143. 10.1093/jmcb/mjy066 30428023 PMC6392100

[B166] Staiger H. Haas C. Machann J. Werner R. Weisser M. Schick F. (2009). Muscle-derived angiopoietin-like protein 4 is induced by fatty acids *via* peroxisome proliferator-activated receptor (PPAR)-δ and is of metabolic relevance in humans. *Diabetes* 58 579–589. 10.2337/db07-1438 19074989 PMC2646056

[B167] Staron R. S. (1997). Human skeletal muscle fiber types: delineation, development, and distribution. *Can. J. Appl. Physiol.* 22 307–327. 10.1139/h97-020 9263616

[B168] Steensberg A. van Hall G. Osada T. Sacchetti M. Saltin B. Pedersen B. K. (2000). Production of interleukin-6 in contracting human skeletal muscles can account for the exercise-induced increase in plasma interleukin-6. *J. Physiol.* 529 237–42. 10.1111/J.1469-7793.2000.00237.X 11080265 PMC2270169

[B169] Sun Z. Singh N. Mullican S. E. Everett L. J. Li L. Yuan L. (2011). Diet-induced lethality due to deletion of the Hdac3 gene in heart and skeletal muscle. *J. Biol. Chem.* 286 33301–33309. 10.1074/jbc.M111.277707 21808063 PMC3190900

[B170] Talbot J. Maves L. (2016). Skeletal muscle fiber type: using insights from muscle developmental biology to dissect targets for susceptibility and resistance to muscle disease. *Wiley Interdiscip. Rev. Dev. Biol.* 5 518–534. 10.1002/wdev.230 27199166 PMC5180455

[B171] Tanianskii D. A. Jarzebska N. Birkenfeld A. L. O’sullivan J. F. Rodionov R. N. (2019). Beta-aminoisobutyric acid as a novel regulator of carbohydrate and lipid metabolism. *Nutrients* 11:524. 10.3390/nu11030524 30823446 PMC6470580

[B172] Tanimura Y. Aoi W. Takanami Y. Kawai Y. Mizushima K. Naito Y. (2016). Acute exercise increases fibroblast growth factor 21 in metabolic organs and circulation. *Physiol. Rep.* 4:e12828. 10.14814/phy2.12828 27335433 PMC4923231

[B173] Tezze C. Romanello V. Sandri M. (2019). FGF21 as modulator of metabolism in health and disease. *Front. Physiol.* 10:419. 10.3389/fphys.2019.00419 31057418 PMC6478891

[B174] Tezze C. Romanello V. Desbats M. A. Fadini G. P. Albiero M. Favaro G. (2017). Age-associated loss of OPA1 in muscle impacts muscle mass, metabolic homeostasis, systemic inflammation, and epithelial senescence. *Cell Metab.* 25 1374–1389.e6. 10.1016/j.cmet.2017.04.021 28552492 PMC5462533

[B175] Tsujinaka T. Fujita J. Ebisui C. Yano M. Kominami E. Suzuki K. (1996). Interleukin 6 receptor antibody inhibits muscle atrophy and modulates proteolytic systems in interleukin 6 transgenic mice. *J. Clin. Invest.* 97 244–249. 10.1172/JCI118398 8550842 PMC507086

[B176] Turner N. Cooney G. J. Kraegen E. W. Bruce C. R. (2014). Fatty acid metabolism, energy expenditure and insulin resistance in muscle. *J. Endocrinol.* 220 T61–T79. 10.1530/JOE-13-0397 24323910

[B177] Tzika E. Dreker T. Imhof A. (2018). Epigenitics and metabolism in health and disease. *Front. Genet.* 9:361. 10.3389/fgene.2018.00361 30279699 PMC6153363

[B178] Villarroya J. Gallego-Escuredo J. M. Delgado-Anglés A. Cairó M. Moure R. Gracia Mateo M. (2018). Aging is associated with increased FGF21 levels but unaltered FGF21 responsiveness in adipose tissue. *Aging Cell* 17:e12822. 10.1111/acel.12822 30043445 PMC6156525

[B179] Volmar C. H. Wahlestedt C. (2015). Histone deacetylases (HDACs) and brain function. *Neuroepigenetics* 1 20–27. 10.1016/j.nepig.2014.10.002

[B180] Walsh M. E. Bhattacharya A. Sataranatarajan K. Qaisar R. Sloane L. Rahman M. M. (2015). The histone deacetylase inhibitor butyrate improves metabolism and reduces muscle atrophy during aging. *Aging Cell* 14 957–970. 10.1111/acel.12387 26290460 PMC4693467

[B181] Wang J. Zhao Y. T. Zhang L. Dubielecka P. M. Zhuang S. Qin G. (2020). Irisin improves myocardial performance and attenuates insulin resistance in spontaneous mutation (Leprdb) mice. *Front. Pharmacol.* 11:769. 10.3389/fphar.2020.00769 32581784 PMC7283381

[B182] Wang S. Wang Y. Zhang Z. Liu Q. Gu J. (2017). Cardioprotective effects of fibroblast growth factor 21 against doxorubicin-induced toxicity *via* the SIRT1/LKB1/AMPK pathway. *Cell Death Dis.* 8:e3018. 10.1038/cddis.2017.410 28837153 PMC5596591

[B183] Watanabe M. Singhal G. Fisher F. M. Beck T. C. Morgan D. A. Socciarelli F. (2020). Liver-derived FGF21 is essential for full adaptation to ketogenic diet but does not regulate glucose homeostasis. *Endocrine* 67 95–108. 10.1007/s12020-019-02124-3 31728756 PMC7948212

[B184] Wei W. Dutchak P. A. Wang X. Ding X. Wang X. Bookout A. L. (2012). Fibroblast growth factor 21 promotes bone loss by potentiating the effects of peroxisome proliferator-activated receptor γ. *Proc. Natl. Acad. Sci. U. S. A.* 109 3143–3148. 10.1073/pnas.1200797109 22315431 PMC3286969

[B185] Willis S. A. Sargeant J. A. Thackray A. E. Yates T. Stensel D. J. Aithal G. P. (2019). Effect of exercise intensity on circulating hepatokine concentrations in healthy men. *Appl. Physiol. Nutr. Metab.* 44 1065–1072. 10.1139/apnm-2018-0818 31453723

[B186] Wu H. Naya F. J. McKinsey T. A. Mercer B. Shelton J. M. Chin E. R. (2000). MEF2 responds to multiple calcium-regulated signals in the control of skeletal muscle fiber type. *EMBO J.* 19 1963–1973. 10.1093/emboj/19.9.1963 10790363 PMC305686

[B187] Xie T. Leung P. S. (2020). Roles of FGF21 and irisin in obesity-related diabetes and pancreatic diseases. *J. Pancreatol.* 3 29–34. 10.1097/JP9.0000000000000039

[B188] Xiong Y. Wu Z. Zhang B. Wang C. Mao F. Liu X. (2019). Fndc5 loss-of-function attenuates exercise-induced browning of white adipose tissue in mice. *FASEB J.* 33 5876–5886. 10.1096/FJ.201801754RR 30721625

[B189] Xu F. Liu J. Na L. Chen L. (2020). Roles of epigenetic modifications in the differentiation and function of pancreatic β-Cells. *Front. Cell Dev. Biol.* 8:748. 10.3389/fcell.2020.00748 32984307 PMC7484512

[B190] Yakabe M. Hosoi T. Akishita M. Ogawa S. (2020). Updated concept of sarcopenia based on muscle–bone relationship. *J. Bone Miner. Metab.* 38 7–13. 10.1007/s00774-019-01048-2 31583540

[B191] Yamauchi T. Kamon J. Waki H. Terauchi Y. Kubota N. Hara K. (2001). The fat-derived hormone adiponectin reverses insulin resistance associated with both lipoatrophy and obesity. *Nat. Med.* 7 941–946. 10.1038/90984 11479627

[B192] Yang Z. Chen X. Chen Y. Zhao Q. (2015). PGC-1 mediates the regulation of metformin in muscle irisin expression and function. *Am. J. Transl. Res.* 7 1850–1859. 26692929 PMC4656762

[B193] Yudkin J. S. Kumari M. Humphries S. E. Mohamed-Ali V. (2000). Inflammation, obesity, stress and coronary heart disease: is interleukin-6 the link? *Atherosclerosis* 148 209–214. 10.1016/S0021-9150(99)00463-3 10657556

[B194] Zhang H. Ren E. Xu R. Su Y. (2021). Transcriptomic responses induced in muscle and adipose tissues of growing pigs by intravenous infusion of sodium butyrate. *Biology (Basel)* 10:559. 10.3390/BIOLOGY10060559 34203067 PMC8234147

[B195] Zhang J. Cheng Y. Gu J. Wang S. Zhou S. Wang Y. (2016). Fenofibrate increases cardiac autophagy *via* FGF21/SIRT1 and prevents fibrosis and inflammation in the hearts of Type 1 diabetic mice. *Clin. Sci.* 130 625–641. 10.1042/CS20150623 26795437

[B196] Zhao Y. T. Wang H. Zhang S. Du J. Zhuang S. Zhao T. C. (2016). Irisin ameliorates hypoxia/reoxygenation-induced injury through modulation of histone deacetylase 4. *PLoS One* 11:e0166182. 10.1371/journal.pone.0166182 27875543 PMC5119735

[B197] Zheng S. L. Li Z. Y. Song J. Liu J. M. Miao C. Y. (2016). Metrnl: a secreted protein with new emerging functions. *Acta Pharmacol. Sin.* 37 571–579. 10.1038/aps.2016.9 27063217 PMC4857552

[B198] Zhu S. Ma L. Wu Y. Ye X. Zhang T. Zhang Q. (2014). FGF21 treatment ameliorates alcoholic fatty liver through activation of AMPK-SIRT1 pathway. *Acta Biochim. Biophys. Sin. (Shanghai)* 46 1041–1048. 10.1093/ABBS/GMU097 25355486

[B199] Zurlo F. Larson K. Bogardus C. Ravussin E. (1990). Skeletal muscle metabolism is a major determinant of resting energy expenditure. *J. Clin. Invest.* 86 1423–1427. 10.1172/JCI114857 2243122 PMC296885

